# Development of Mixed metal Metal-organic polyhedra networks, colloids, and MOFs and their Pharmacokinetic applications

**DOI:** 10.1038/s41598-017-00733-4

**Published:** 2017-04-11

**Authors:** Nazir Ahmad, Hussein A. Younus, Adeel H. Chughtai, Kristof Van Hecke, Muhammad Danish, Zhang Gaoke, Francis Verpoort

**Affiliations:** 1 Laboratory of organometallics, catalysis and Ordered Materials, State Key Laboratory of Advanced Technology for Materials Synthesis and Processing, Wuhan University of Technology, Wuhan, 430070 China; 2 School of Resources and Environmental Engineering, Wuhan University of Technology, Wuhan, 430070 China; 3 Chemistry Department, Faculty of Science, Fayoum University, Fayoum, 63514 Egypt; 4 Institute of Chemical Sciences, Bahauddin Zakariya University, Multan, 60800 Pakistan; 5 Department of Inorganic and Physical Chemistry, Laboratory of Organometallic Chemistry and Catalysis, Ghent University, Krijgslaan 281 (S-3), 9000 Ghent, Belgium; 6 Department of Chemistry, University of Gujrat, Gujrat, 50700 Pakistan; 7 National Research Tomsk Polytechnic University, Lenin Avenue 30, Tomsk, 634050 Russia; 8 Ghent University Global Campus Songdo, 119 Songdomunhwa-Ro, Yeonsu-Gu, Incheon 406-840 South Korea

## Abstract

The coordination networking of discrete metal-organic polyhedra (MOPs) involving different ligands as well as metals is a challenging task due to the features of limited solubility and chemical stability of these polyhedra. An unusual approach, ligand-oriented polyhedral networking *via* click chemistry and further metal coordination is reported here. An alkyne decorated Cu(II)-MOP self-catalyzes the regioselective click reaction (1,3-dipolar cycloaddition) using azide-functionalized ligands under unconventional reaction conditions. Introducing new metal ions, M(II), interlinks the carboxylic groups on the MOP surfaces creating coordination networks. On the other hand, exposure of the respective individual ligand components in the presence of Cu(II) promotes an *in-situ* click reaction along with metal coordination generating a new 3D-framework. These materials demonstrated a high drug hosting potential exhibiting a controlled progressive release of anticancer (5-flourouracil) and stimulant (caffeine) drugs in physiological saline at 37 °C. These innovative and unconventional MOP networks provide a significant conceptual advance in understanding.

## Introduction

The strategy to utilize metal-organic polyhedra (MOPs) as supramolecular monomers for porous coordination polymeric networks is unique and of interest to all researchers across the chemical sciences^1–5^. The functional ligands decorating the MOP surface are used to introduce further functionalization^6–11^. This is achieved by exploiting the stability and solubility of MOPs that may have potential to coordinate in the possible dimensions. The post-synthetic modification (PSM) permits characterization of the heterogeneity in a given material. There are different forms of the ordered heterogeneity including mixing of new (a) organic linkers, (b) metals, or (c) both within the same network backbone^10^. In order to achieve the PSM of MOPs the main requirements are solubility and stability. PSM in metal-organic materials is particularly advantageous for the formation of such materials that cannot be obtained using established methodologies^1, 11^.

To extend the nanometer sized MOPs using a coordination process, two possible options are available either through the connection of *vertices* (metallic nodes)^12–14^ or *edges* (ligand). At the MOP periphery, as compared to the bridging ligands, metal sites are blocked by coordinated solvent(s). Furthermore, MOPs have a higher number of ligand bridges exposed at the surface than that of the metallic nodes (*e.g*. M_2_(COO^−^)_4_ or the coordination spheres having cage formula M_*n*_L_2*n*_)^15^. Therefore, introducing a new covalent functionalization containing electron (*e* ^*−*^) donor group(s) over the supramolecular cages in the presence of M(II) ions can interconnect these precursors giving rise to a novel porous coordination network. This approach would create a network in which higher number of MOPs may get interlinked during polymerization. Here, the post-synthetic modification to the MOP structure leading towards an ordered heterogeneity to develop a MOP framework by mixing various metallic nodes as well as organic linkers was performed. Hence, materials based on unconventional supramolecular coordination networking can be engineered.

## Results and Discussion

**Figure 1:**
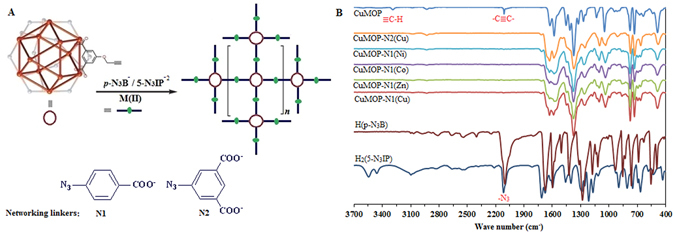
(**A**) Post-synthetic modification of alkyne-functionalized CuMOP into its extended networks using azide substituted organic bridging linkers (*4*-azidobenzoate (*4*-N_3_B^−^) = N1 and 5-azidoisophthalate (5-N_3_IP^−2^) = N2) *via* click reaction and their further coordination with metal(II) ions. (**B**) FTIR spectra of the H(*p*-N_3_B), H_2_(5-N_3_IP), CuMOP, and polymeric networks.

As a proof-of-concept, we selected a nanocage [Cu_24_(5-prop-2-ynoxyisophthalate)_24_(H_2_O)_24_] (CuMOP) decorated with alkyne groups to be converted by “click chemistry” in to *e* ^−^ donors (Figs S1 and S2). CuMOP is soluble in high-boiling organic solvents *e.g*., *N*, *N*-dimethylformamide (DMF) and remains stable (for more details see in the supplementary information Figs S4–S8)^16^. The surface functional organic linkers on the MOP cage provide a forum of an efficient *e* ^−^ donor sites for metal (M) complexation. In the case of organic bridging linker N1, the CuMOP surface contains 24 carboxylic (–COOH) groups which become activated for further coordination with the addition of a metal (Fig. 1A). When linker N2 is used, the number of the carboxylic sites at the surface of MOP entities is double (48). As a result, MOP polymeric networks with the common compositions MOP-N(M)s are generated, where N is a networking linker.

**Figure 2:**
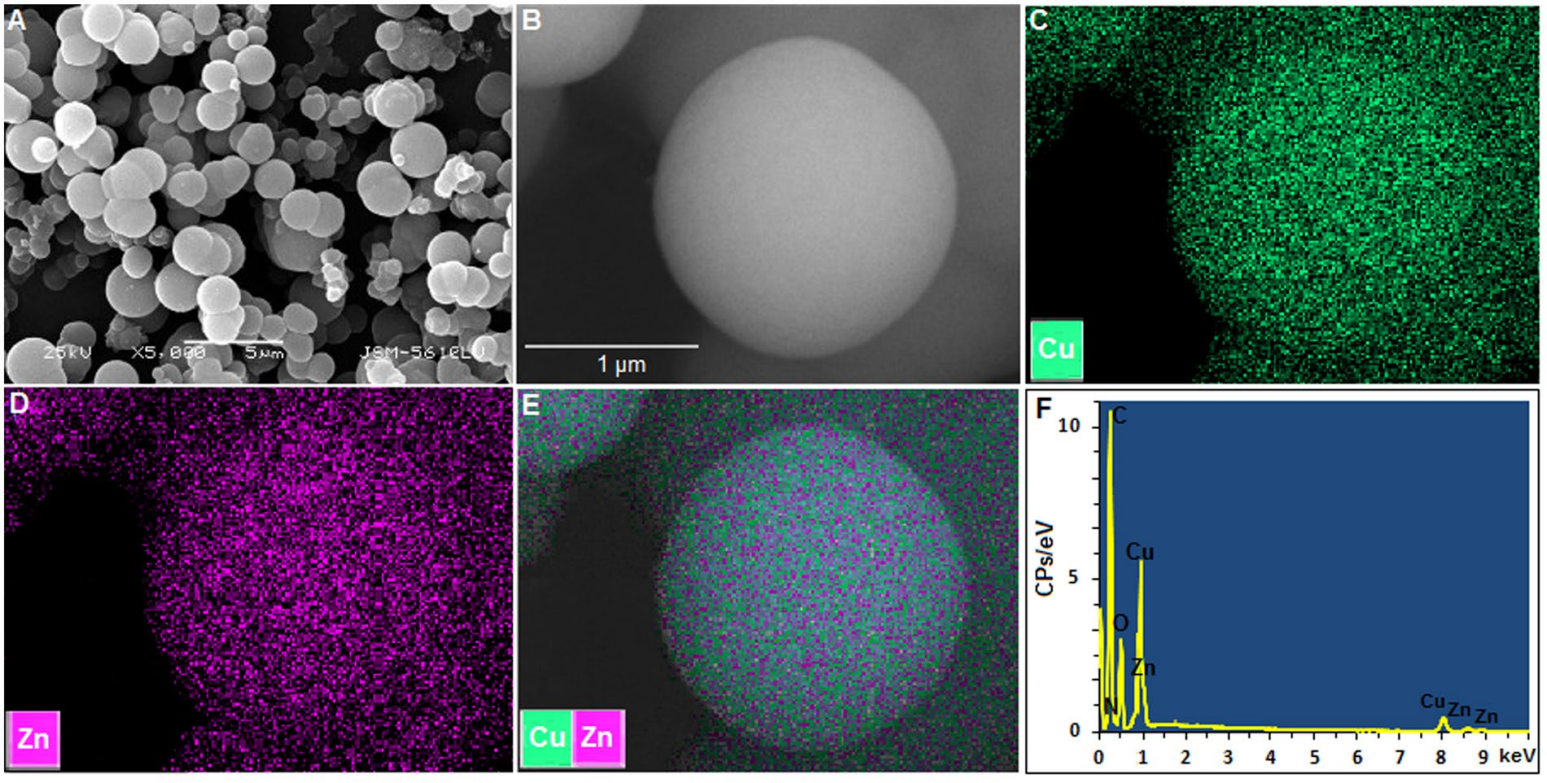
Field emission scanning electron microscopic (FE-SEM) images (**A** and **B**), energy-dispersive spectroscopic (EDS) mapping analysis for copper (**C**), zinc (**D**), and their overlay mapping (**E**), and EDS compositional analysis of the CuMOP-N1(Zn) obtained from overall elemental mapping analysis (**F**).

Fourier transform infrared (FTIR) analysis of the products showed the disappearance of azide and alkyne groups (Fig. 2B) indicating that CuMOP underwent Huisgen cycloaddition between its alkyne and azide functionalities without conventional click conditions. The MOPs were clicked into their coordination networks CuMOP-N(M)s as confirmed from the FTIR spectra. FTIR spectra of the products showed that all of the alkyne and azide functionalities were converted into triazole bridging groups. FTIR comparison analysis of the CuMOP, HN1/H_2_N2, and the CuMOP-N(M)s is presented, and it is obvious that all of the alkyne groups around the MOP cage units are clicked into azide groups, as the vibrational bands of alkyne (≡C–H at 3290 cm^−1^ and –C≡C– at 2119 cm^−1^) in CuMOP and azide (–N_3_ at 2115 cm^−1^) of N1 and N2 linkers are fully consumed in the clicked products.

### Self-catalysis for highly regioselective formation of 1*H*-1,2,3-triazole

The catalytic nature of the click reaction is confirmed by the control (blank) reaction, where mixing of H_2_(*5*-N_3_IP) with *5*-prop-*2*-ynoxyisophthalic acid (H_2_ *5*-PIP) in the absence of CuMOP under the same conditions does not proceed the click reaction (Fig. S9). Therefore, in the reaction protocol (Scheme in Fig. 1) CuMOP serves as a catalyst by self-catalyzing its surface alkyne groups with azide linkers into triazole bridging groups. The catalytic role of the MOP precursor is advantageous as compared to the use the of conventional click catalyst providing the effectiveness of the reaction methodology. To check that whether CuMOP serves as a catalyst of the click reaction or it decomposes under the reaction conditions to give Cu^2+^ ions which might serve as catalyst. Therefore, the stability of the CuMOP was checked at room temperature and under the reaction conditions. And, it was found stable without any detectable decomposition. Cu-Cu paddle-wheels were maintained in the MOP structure and did not generate Cu^2+^ ions in the solution (only the peak of Cu-Cu paddle-wheel appeared at 718 nm, (Figs S4 and S8), otherwise for the free Cu^2+^ ions in the solution a peak should appear at 800 nm)^16^. Moreover, the progress of the click reaction was similar in case of synthesis of other materials *i.e*., CuMOPN1-(M) where M is Zn(II), Co(II), and Ni(II) (Fig. 1). These observations excludes the possible contribution of the Cu^2+^ ions to drive the click reaction and supporting the catalytic role of the MOP for the click reaction.

To follow the click reaction, ^1^H nuclear magnetic resonance (NMR) measurements of the ligand produced from the degradation of the coordination products were analyzed. The ligands (obtained after the degradation process) contained the triazole ring with a highly regioselective formation of 1*H*-1,2,3-triazole moiety deriving from alkyne and azide functionalities (Figs S10 and S11). This result, *i.e*. the formation of 1*H*-1,2,3-triazole moiety, is more critical than the formation of 1*H*-1,2,4-triazole group because the corresponding link will be more bent towards the surface of CuMOP which could cause stereochemical hindrance for the terminal –COOH groups and probably block the reaction sites. Whereas, in the former case the linker is less bent and the sequence of (carboxyphenyl)-1*H*-1,2,3-triazol)methoxy is oriented outward from the surface of CuMOP providing no steric hindrance for metal coordination. Hereafter, the interconnections of CuMOPs through metal coordination supported links become quite feasible. The networking between CuMOPs resulted in the hetero-metallic composition formulated by the various combinations of transition metals. Hence, a coordination network is created, where nanosized polyhedral cages are linked with each other through their organic bridging linkers oriented at surfaces/edges.

The number of carboxylic sites around a CuMOP connected with similar sites of other CuMOPs based on the above description is calculated from elemental analysis measurements. The observed values of CHN (Table S1) and inductively coupled plasma (Table S2) analysis fit best to the description that 12 coordination sites around the CuMOP contribute to the self-networking keeping the rest as uncoordinated. This may be due to the large size of the MOP and stereochemical requirements demanded for the coordination driven self-assembling of polyhedral packing resulting in this arrangement. Therefore, 12 CuMOPs surround a CuMOP and so on it grows in a network fashion. In the framework material, the uncoordinated sites around MOPs develop interactions with the entrapped solvent molecules. For CuMOP-N1(M) there are 12 free carboxylic groups, whereas there are 36 of such sites in case of CuMOP-N2(M). Therefore the later, exclusively involves weak interactions with solvent molecules and generates a colloidal solution. The analysis of the supernatants resulting from the degradation experiments for CuMOP-N2(Cu) using high resolution electrospray mass spectrometric (ESI-MS) showed species corresponding to [Cu_8_L^4^-4H + H_2_O]^+^, [Cu_4_L^4^-4H]^+^, [Cu_3_L-4H + Na]^+^, and [L^4^ + H_2_O)]^+^ where L is 5-(4-((3,5-dicarboxyphenoxy)methyl)-1*H*-1,2,3-triazol-1-yl)isophthalate (Fig. S12).

### Further coordination of MOPs with more/different metals

This unconventional reaction strategy is attractive for the synthesis of different combinations of various metal centers (Cu:Cu, Cu:Zn, Cu:Co, and Cu:Ni *etc*.) within the same network. Also, the population of metal connecting centers around the MOP entities can be controlled based on the selected metals’ coordination behavior to carboxylic groups. For example, when metallic paddlewheel (PW) clusters (*e.g*., copper(II) and Zn(II) dimer *etc*.) are involved, then it will give rise to a higher metal contents as compared to the metallic nodes having the mononuclear coordination behavior with the carboxylic group (*e.g*., cobalt(II) and Ni(II) *etc*.) (see Table S2). This type of metal heterogeneity is beneficial for the integration of different properties. This also gives a particular control to improve any given property (*e.g*. low temperature magnetism^17–19^ Figs S13 and S14).

Field emission scanning electron microscopic (FE-SEM) images revealed the spherical morphology of the CuMOP-N1(Zn) network. The overall shape of the network structure constructed by spherical MOP nanomaterials is also of spherical dimensions. These micro spherical shapes are obvious a result of self-assembling of the nanosized MOP spheres. The heterometallic-compositions and the symmetric distributions of CuMOPs are further substantiated by elemental mappings using energy-dispersive spectroscopy (EDS) (Fig. 2).

**Figure 3:**
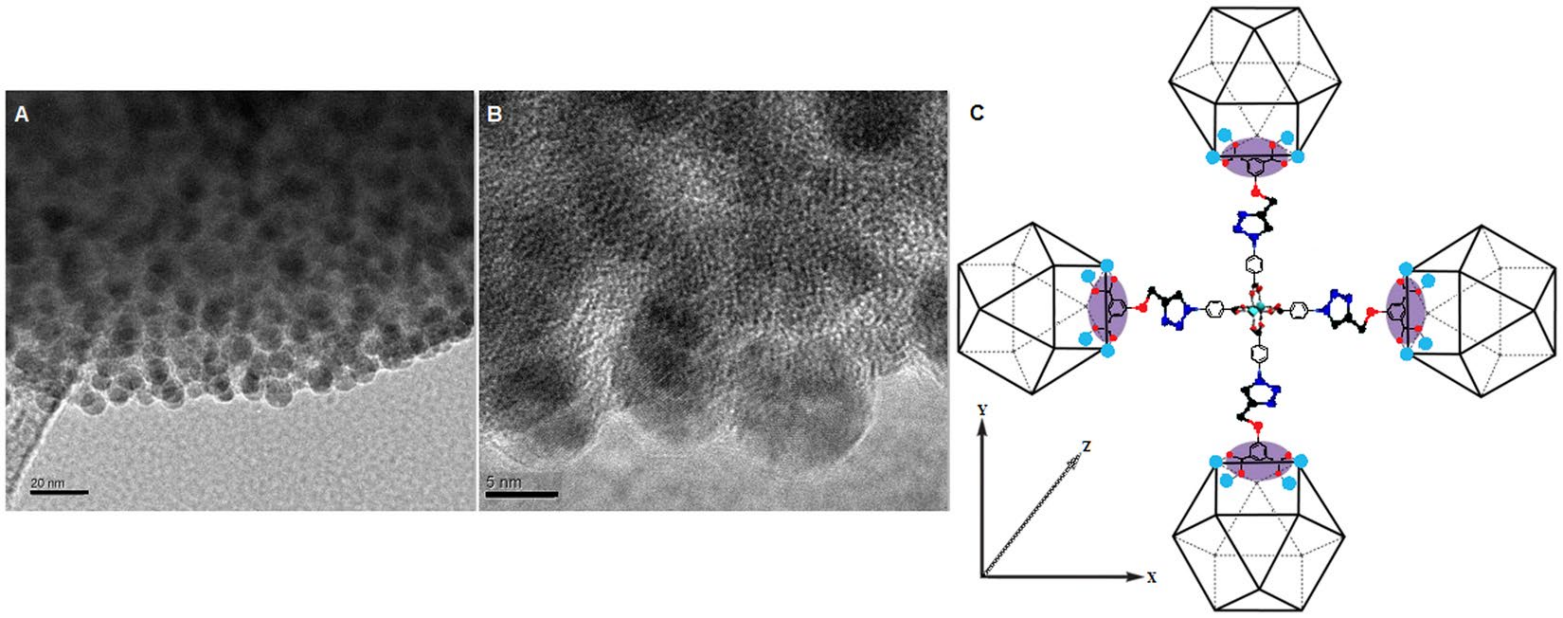
HR-TEM images of the CuMOP-N1(Cu) polymer (**A**) and (**B**) showing the regular distribution of spherical cages within the extended coordination network, and (**C**) the ligand oriented stepwise construction of metal-organic extended network based on MOPs using their *edges* (ligands) *via* click reaction and further metal coordination.

According to the EDS analysis, the overlay of these atom signals shows that CuMOPs are uniformly hybridized to form the 3D microspherical balls without any significant aggregation. This structural feature is caused by covalent and coordination based connections of nanocages triggered by the 3D nanoscale geometry. Similar results are obtained when Cu(II), Co(II), and Ni(II) are used for coordination networks (Figs S15 and S16). These microspheres are further analyzed by high-resolution transmission electron microscopy (HR-TEM). Notably, the existence of the nanocage entities of CuMOP in the overall structural framework is clearly visible (Figs 3 and S17).

This evidences the ligand-oriented stepwise construction of a metal-organic extended network based on MOPs using their *edges* (ligands) *via* click reaction and further metal coordination. The average size of the constructing unit of the network is measured as approximately 5 nm in diameter. This is almost the same size dimension of the diameter of the discrete polyhedral MOP particles in the solution of DMF^15^. Similarly, TEM of CuMOP-N2(Cu) shows that it has a sponge-like morphology and also its basic construct is MOP (Fig. S18). The CuMOP can be obtained in the crystalline state (Fig. S2), whereas the CuMOP polymeric network are not crystalline as the PXRD (Fig. S19) indicate that these are amorphous.

The thermal behavior of the MOP networks was examined by thermogravimetric (TG) analysis (Fig. S20). A multistage degradation pattern was observed for all compounds. The thermal behavior of the given samples is analogous, because of the similarity of the overall structure. A general degradation pattern is described herewith. An initial weight loss (<7.5%) till 100 °C is observed which is assigned to the loss of water molecules, followed by a loss of 11–15 wt.% associated with the extrusion of the trapped solvent (DMF) in the cavities along with the removal of the axially coordinated solvent molecules which completes around 250 °C. Afterwards, the decomposition steps characterize the collapse of the main structure *i.e*. overall backbone of the framework which is mainly composed of CuMOP. This is well in agreement with the reported fact that the MOP architecture remained stable up to 250 °C^16^. The main breakdown of the structure is discriminated by the sharp inflection in the TG curve after 250 °C corresponding to the disintegration of the bridging organic linker and its bonding with the metallic nodes. This TG region is pretty characteristic with the multiple TG steps (2–4) as seen in the Fig. S14, inferring the monometallic and bimetallic (Cu/M, where M is Co, Ni, and Zn) nature of the networks requiring different energies of activations for thermolysis. The residual material, over 15% was obtained till 1000 °C, is expected to be metallic oxide.

### Unconventional synthesis of a 3D Cu(II)-MOF

**Figure 4:**
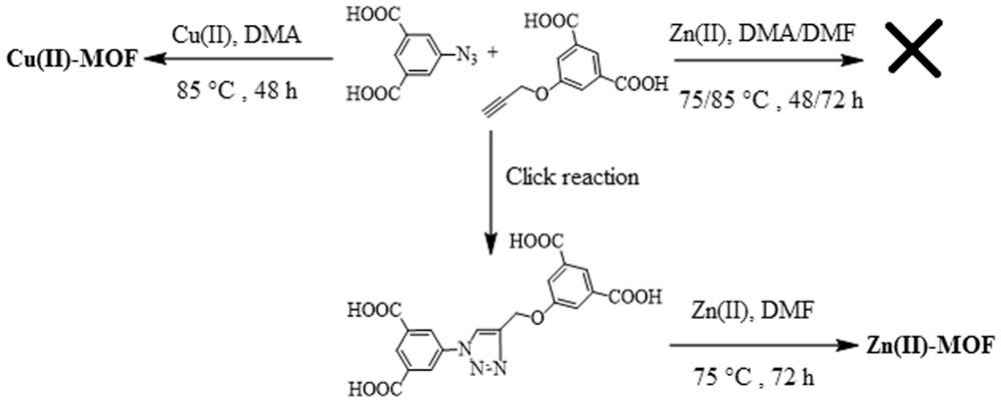
Metal-organic frameworks based on diisophthalate bridging linker.

Following a similar reaction protocol for click, synthesis of the metal-organic frameworks (MOFs) based on alkyne-azide clicked di-isophthalate linker formed *in-situ* is tested (Fig. 4). Generally, the ordinary synthesis of MOFs follows the preparation, purification and characterization of the designed ligands. This routine is at its peak and is affording a limitless number of MOF structures. Here an unconventional approach to generate MOF structures is described, in which the metal-organic self-assembling along with *in-situ* formation of the organic linker is applied. This involves the integration of the synthetic steps into one stride which is rather advantageous as compared to the conventional methodology. Therefore, we idealized that isophthalic acids bearing azide (H_2_5-N_3_IP) and terminal alkyne (H_2_5-PIP) with a slight excess of copper(II) nitrate under solvothermal conditions would simultaneously generate 1*H*-1,2,3-triazole and MOF. The mechanistic details may be deciphered by the simultaneous occurrence of the events *i.e*., deprotonation of the acid groups followed by the formation of PW SBUs, and their catalytic role for the click reaction. As a result, blue block crystals (Fig. S21) of porous Cu-MOF suitable for single crystal X-ray analysis are obtained.

**Figure 5:**
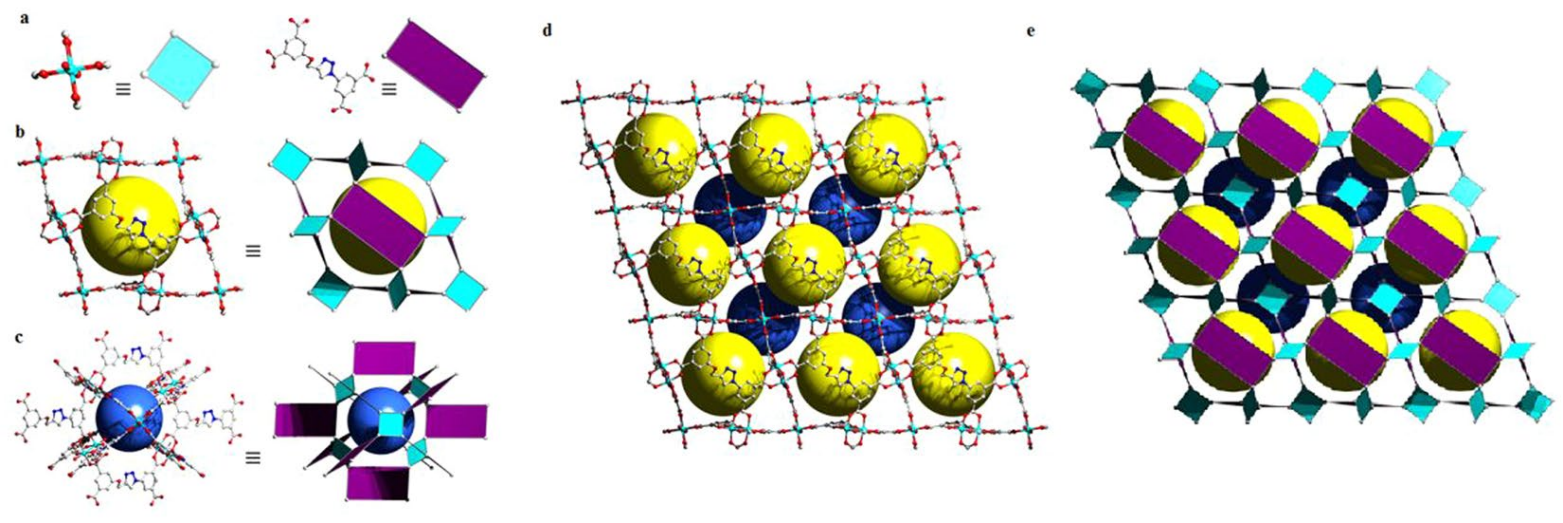
Representation of (**A**) the square Cu_2_(CO_2_)_4_ SBU’s and rectangular L^4−^- SBU’s; two different polyhedral pores (**B**) and (**C**) along with the polyhedral packing of these pores in the 3D framework of Cu-MOF (**D**) and (**E**) with yellow and blue spheres embody the spheres occupying the cavities, taking into account the interior VDW radii. Hydrogen atoms are omitted for clarity.

Single crystal X-ray analysis revealed that compound Cu-MOF crystallized in the trigonal space group R-3m and features a 3D porous MOF structure, characterized by the framework formula [Cu_2_(L)(H_2_O)_2_]_*n*_. The asymmetric unit contains one fourth of an L (5-(4-((3,5-dicarboxyphenoxy)methyl)-*1H*-1,2,3-triazol-1-yl)isophthalate) ligand (the total ligand is generated by a crystallographic mirror plane and a 2-fold rotation axis), one Cu(II) ion with one coordinated water molecule (both on a special position, *i.e*. a mirror plane, with 0.5 occupancy factors). The implied crystallographic symmetry causes the oxymethyl-1*H*-1,2,3-triazole moiety to be four-fold disordered in the crystal structure (Fig. S22). In the crystal structure, typical Cu_2_ PW clusters are formed between two neighboring Cu(II) ions, which are bridged by four μ_2_-η^1^:η^1^ coordinating carboxylate groups from four different L ligands, leading to square-planar 4-coordinated PW dimer nodes, with a Cu-Cu distance of 2.6696(9) Å. The octahedral coordination environment of each Cu(II) ion is completed by one coordinated H_2_O molecule. The standard net topology is found 4,8-coordinated 4,8T24, with a point symbol of {4^4^.6^16^.8^8^}{4^6^}_2_ for the net (Figs S23 and S24). The framework further consists of 3-coordinated nodes corresponding to benzene rings of each of the L ligands. The underlying net of the framework thus is 3,4-coordinated with a **fof** topology, which is considered a derived **nbo** net (Fig. S26). The Cu_2_(CO_2_)_4_ SBUs can be considered as a square secondary building unit (SBU), while rectangular SBU’s are formed by the L^4−^ ligands (Fig. 5A). The carboxylate groups of the L^4−^ ligands are almost perfectly co-planar with the phenyl rings (dihedral angle of 2.3°), making the square SBU’s to be nearly orthogonal to the rectangular SBU’s. The ligand phenyl rings are almost parallel (dihedral angle of 12.4°). This arrangement features two kinds of pores, similar to the previously reported framework, with 3,3′,5,5′-biphenyltetracarboxylate as the ligand^20^. The first pore (Fig. 5B) is defined by six rectangular SBU’s (representing the faces of a rhombohedron) with a pore diameter of ∼12.3 Å (taking the van der Waals radii into account), with a spherical volume of 974 Å^3^ while the second pore (Fig. 5C) is defined by six square SBU’s and a diameter of ∼12.0 Å (terminal waters on the Cu_2_ clusters not taken into account), with a spherical volume of 905 Å^3^. The total potential solvent accessible void volume was calculated to be 8622.7 Å^3^ (68.7% of the unit cell volume) using PLATON^21^.

Similar to Cu-MOF, the synthesis of Zn-MOF was also endeavored. Unfortunately, it failed to be generated by the *in-situ* formation of the organic linkers revealing the importance of the catalytic role of copper(II) for the click reaction (Fig. 4). Then, in order to confirm the synthesis of the respective MOF (Fig. S27) pre-clicked ligand H_4_L was employed (Fig. 4). Zn-MOF crystallized in the monoclinic chiral space group *P*2_1_ and forms a 3D MOF structure, with the framework formulated as [Zn_2_(L)(DMF)_3_]_*n*_, (see supplementary information Figs S28–S37).

### Drug delivery

**Figure 6:**
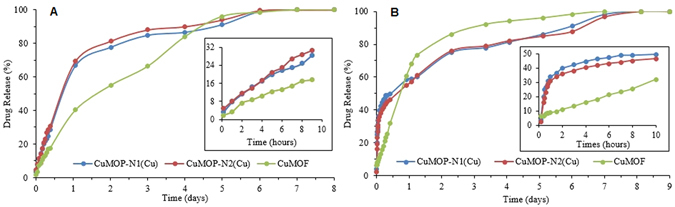
Release profiles of the drugs (**A**) 5-flourouracil (5-FU) and (**B**) caffeine (CAF) from the drug-loaded materials. Loading contents for CuMOP-N1(Cu): 32.21% (5-FU) and 39.74% (CAF); CuMOP-N2(Cu): 32.37% (5-FU) and 35.58% (CAF); Cu-MOF 19.49 (5-FU) and 23.76% (CAF).

The Zn-MOF (Figs S31 and S32) is a different structure also prepared in a different manner, but to some extent MOP coordination networks can be compared with the Cu-MOF. In Cu-MOF, the two roughly spherical pores are arranged rhombohedrally, where each of which surrounds the other by eight respective pores creating an interconnected porous network. There are three of each pores (total number is six) per unit cell (Fig. 5). But in the former case (MOP-N(M)), there are approximately twelve coordinated sites around each MOP and thereby supramolecular pores have been interconnected. Therefore, the inherent porosity and large cage size of the MOP as precursor for such kind of coordinating network would be useful for the multifold encapsulation and then release of small drug molecules. Pharmacokinetic studies are performed to investigate the drug release potential of the MOP networks and are also compared with the Cu-MOF. The loading of 5-fluorouracil (5-FU) and caffeine (CAF) for MOP networks was observed to reach almost 40% (loading contents for CuMOP-N1(Cu) are 32.21% and 39.74%, whereas, for CuMOP-N2(Cu), 32.37% and 35.58%, respectively), which is much higher than the Cu-MOF *i.e*., 19.49 and 23.76%, respectively. The drug release experiments are carried out by dialyzing the drug-loaded materials in the simulated body fluid (PBS pH 7.4) at 37 °C. The observed release profiles of 5-FU and CAF (Fig. 6) are progressive without any burst effect^22^. Both types of materials *i.e*., MOP polymeric network and MOF in buffer solution were found stable (Figs S42 and S43) supporting the mechanism of progressive release of drugs.

The complete discharge of the drug happened within the span of a week. Two stages associated to the drug delivery could be distinguished. Around 15–50% of the loaded contents were released in the first period of 9–10 h (in set plots of Fig. 6) followed by the 85–50% released in the later period of 7–8 days. The encapsulated drug has host-guest interactions inside the cage cavities (H-bonding, π-π, *etc*.) and intermolecular interactions for the packed drug molecules. These interactions may account for the different regimes during the release. The initial stage may arise from the weak intermolecular interactions and require less time and *vice versa* for the later. The delivery performance of the MOP networks is quite comparable with the MOF materials while keeping the higher drug loading capacity than MOFs. These results of drug loading and release are remarkable, because the precursor MOP has been reported previously for the loading and release of 5-FU (4.38%, 8 h)^7^ and CAF (4.85%, 7 h)^16^. Similarly, CuMOP-N(M)s materials offer higher efficiency than the reported MOF for the respective studies of 5-FU, where drug loading was 23.76% and burst release was completed in 10 h^13^.

## Conclusions

All results discussed above evidently demonstrate the ligand-oriented metal-organic polyhedral networking by *in-situ* click and further coordination for controlled progressive release of encapsulated drugs. The features of the work contain unprecedented results *e.g*., (i) self-catalysis of the coordination cage creating new reaction sites for (ii) further coordination with more/different metals leading to exceptional coordination networks. The general scheme of the reaction afforded solids as well as colloidal solutions based on the number of electron donor sites around the cage. Additionally, by changing the addition sequence of the same precursors, the *in-situ* generated ligand forms the metal-organic framework selectively to the copper(II). The self-catalyzed click reaction by the coordination cages is due to the presence of copper(II)-carboxylate motifs as it is proved by the synthesis of 3D Cu(II)-MOF based on the *in-situ* generated di-isophthalate linker. Apart from the monometallic MOFs, several multi-metallic combinations during the supramolecular networking can be housed. The metallic heterogeneity is beneficial for the assimilation of different properties. This also gives a particular control to improve any given property *e.g*. low temperature magnetism. Current methodology introduced new exiting materials exploiting the coordination of supramolecular self-assemblies. Clearly, designing a MOP based network or other composites with higher density of MOPs will substantially improve the overall loading and slow release performance of particular molecule, *e.g*., 5-FU and CAF in CuMOP-N(M)-based systems presented here.

In summary, our approach for the material design and synthesis is potentially applicable to achieve the supramolecular coordination and the effective use of these materials in the slow drug release in pharmacokinetics. We believe that the concept of the *in-situ* click and coordination driven self-assembling of the supramolecular self-assemblies and unconventional synthesis of established MOF structures will not only lead to a new approach for the storage and delivery of drug molecules, but will also open up a new field of study exciting with coordination structures based on supramolecular precursors and applications in the field of sorption and catalysis.

## Methods

### Material synthesis

The general procedures for the material’s synthesis are described here, whereas the full details are given in the supplementary information. CuMOP, organic bridging linker (*i.e*., azide substitute d benzoic or isophthalate *4*-N_3_B^−1^ (N1) or *5*-N_3_IP^−2^ (N2), respectively), and metal (Cu(II), Zn(II), Co(II), or Ni(II) *etc*.) nitrate are reacted solvothermally in DMF/EtOH (v/v, 2:1) by subjecting to heat in a Teflon lined autoclave for 35 h at 90 °C. Networking linker N1 afforded powdered materials CuMOP-N1(M), whereas, a blue colloidal suspension CuMOP-N2(Cu) was achieved from the linker N2. For the *in-situ* synthesis of Cu-MOF, the alkyne and azide substituted diisophthalic acid linkers (H_2_-5PIP and H_2_-N_3_IP) and copper nitrate in DMA are mixed with few drops of H_3_BO_4_ and heated to 85 °C for 2 days. Whereas in the case of Zn-MOF, ligand 5-(4-((3,5-dicarboxyphenoxy)methyl)-1*H*-1,2,3-triazol-1-yl)isophthalic acid (H_4_L) and zinc nitrate are mixed in DMF and subjected to solvothermal heating at 75 °C for 3 days.

### X-ray single-crystal diffraction

For the structure of compound Cu-MOF, X-ray intensity data were collected on a Agilent Supernova Dual Source (Cu at zero) diffractometer equipped with an Atlas CCD detector using CuKα radiation (λ = 1.54178 Å) and ω scans. For the structure of compound Zn-MOF, X-ray intensity data were collected on a Bruker diffractometer equipped with an APEX II CCD detector using MoKα radiation (λ = 0.71073 Å) and φ and ω scans. For Cu-MOF, the images were interpreted and integrated with the program CrysAlisPro (Agilent Technologies). For Zn-MOF, the images were interpreted and integrated with the program SAINT from Bruker. Using Olex2^23^, the structures were solved by direct methods using the ShelXS structure solution program and refined by full-matrix least-squares on F^2^ using the ShelXL program package^21^. For both structures, non-hydrogen atoms were anisotropically refined and the hydrogen atoms in the riding mode and isotropic temperature factors fixed at 1.2 times U(eq) of the parent atoms. The contribution of heavily disordered solvent molecules was taken into account and suppressed using the SQUEEZE procedure in PLATON^24^. CCDC-1418777 and CCDC-1418778 contain the supplementary crystallographic data for Cu-MOF and Zn-MOF respectively.

### Drug loading and release experiments

Loading of the 5-fluorouracil and caffeine was carried out by stirring the 100 mg drug dissolved in the methanol (30 mL) with 50 mg dehydrated materials (100 °C under vacuum for 12 h) for three days. The materials were separated and washed with methanol by centrifugation and dried in vacuum oven at 50 °C for 12 h. The adsorbed amount of drugs was measured by UV/Vis absorption spectroscopy. The drug-loaded samples are subjected to the release experiments. In each case, 15 mg of the material is dialyzed against 100 mL of PBS (pH 7.4) at 37 °C with gentle stirring in semi-permeable dialysis bags (molecular weight cut-off, MWCO = 1000 for 5-FU and 2000 for CAF). At different time intervals, 3 mL of the solution was taken out for analysis. The contents of the 5-FU and CAF in the release media were monitored by fluorometry at 453 nm and UV/Vis absorption analysis, respectively.

## Electronic supplementary material

Supplementary information
