# Supplementary material for: Development of Mixed metal Metal-organic polyhedra networks, colloids, and MOFs and their Pharmacokinetic applications
Source: Sci Rep. 2017 Apr 11;7:832. doi: 10.1038/s41598-017-00733-4 (PMC5429760; doi:10.1038/s41598-017-00733-4)
Supplement: Supplementary file 1 — Supplementary information [file 41598_2017_733_MOESM1_ESM.pdf]

Supplementary Information:

Development of Mixed metal Metal-organic polyhedra networks, colloids, and MOFs and their Pharmacokinetic applications

Nazir Ahmad,[1,2] Hussein A. Younus,[1,2,3] Adeel H. Chughtai,[4] Kristof Van Hecke,[5] Muhammad Danish,[6] Zhang Gaoke,[2] Francis Verpoort[1,*,7,8]

[1] Laboratory of organometallics, catalysis and Ordered Materials, State Key Laboratory of Advanced Technology for Materials Synthesis and Processing, Wuhan University of Technology, Wuhan 430070, China. *e-mail: Francis.verpoort@ugent.be

[2] School of Resources and Environmental Engineering, Wuhan University of Technology, Wuhan 430070, China

[3] Chemistry Department, Faculty of Science, Fayoum University, Fayoum 63514, Egypt.

[4] Institute of Chemical Sciences, Bahauddin Zakariya University, Multan 60800, Pakistan.

[5] Department of Inorganic and Physical Chemistry, Laboratory of Organometallic Chemistry and Catalysis, Ghent University, Krijgslaan 281 (S-3), 9000 Ghent, Belgium.

[6] Department of Chemistry, University of Gujrat, Gujrat 50700, Pakistan.

[7] National Research Tomsk Polytechnic University, Lenin Avenue 30, Tomsk-634050, Russia.

[8] Ghent University Global Campus Songdo, 119 Songdomunhwa-Ro,Yeonsu-Gu, Incheon 406-840, South Korea.

Contents

1. Materials and Methods
2. Stability of CuMOP
3. NMR and ESI-MS spectra
4. CHN analysis
5. ICP analysis
6. Magnetic measurements
7. SEM, EDS, TEM, PXRD, TGA analysis
8. Single crystal X-ray diffraction
9. FTIR and TGA of MOFs
10. Stability of the materials in buffer solution

1. Materials and Methods:

1.1. Materials

All reagents and solvents employed were commercially available (Aladdin) and used without further purification. Samples were prepared using solvothermal methods from solutions of the starting materials, which included the nitrate salt of the metal ions, *5*-substituted-*1*,*3*-benzene dicarboxylate (H_2*5*-R-BDC) ligand. The procedures to synthesize *5*-prop-*2*-ynoxyisophthalic acid ($H_2$5-PIP)[1], and CuMOP and 4-azidobenzoic acid (H*4*-B), and 5-azidoisophthalic acid (H_2*5*-N_3IP) [2] were adopted as described in literature.

1.2. Instruments

Fourier transform infrared (FT-IR) spectra were recorded on a Perkin-Elmer Spectrum One spectrometer. Powder X-ray diffraction (XRD) analysis performed on a D8 Advance diffractometer (Bruker). UV/Vis spectra were recorded on a Shimadzu UV-1800 spectrophotometer at room temperature. Fluorescence analysis were taken on Perkin Elmer Light Spectrometer LS-45. ^{1}H-NMR spectra were recorded at Bruker 500 MHz NMR spectrometer. Mass spectra were obtained with a nano electrospray ionization-quadrupole time-of-flight mass spectrometer (ESI-Q-TOF MS) (Bruker) in the positive mode. High resolution mass spectrum were collected on a Bruker Daltonics Solarix 7.0 Tesla Fourier Transform Mass Spectrometer. Elements in the sample were analyzed by CHNS analyzer (Vario EL Cube) and inductively coupled plasma (ICP) emission spectrometer (ICP 6000). Metal mapping analysis by Field Emission Scanning Electron Microscope (FE-SEM) images and SEM-EDS data were taken on a ZEISS ULTRA PLUS-43-13 on connection to an Energy Dispersive Spectrometer (OXFORD X-Max 50). The acceleration voltage was 15 kV. The shape and microstructure was analyzed by scanning electron microscopy (SEM) (JSM-5610LV). High-resolution transmission electron microscopy (HRTEM) imaging was recorded with a HRTEM (JEM2100F) at an accelerating voltage of 200 kV. Temperature dependent and field dependent Magnetic analysis are performed by physical property measurement (PPMS-9, quantum design). Thermogravimetric (TG) analysis of the samples was carried out using a Netzsch STA 449C Jupiter Aeolos (German) coupled to mass spectrometer with a heating rate of 10 °C/min under nitrogen atmosphere. Single crystal X-ray data was collect

on Agilent Supernova Dual Source (Cu at zero) diffractometer equipped with an Atlas CCD detector using CuKα radiation (λ = 1.54178 Å) and ω scans.

1.3. Synthesis of CuMOP-N(M)s

The homogeneous solutions of CuMOP (8.3×10^{-3} mmol, 62.5 mg dissolved in 6 mL DMF/EtOH (v/v, 2:1)), *4*-azidobenzoic acid (0.2 mmol, 32.6 mg dissolved in 3 mL DMF/EtOH (v/v, 2:1)), and $Cu(NO_3)_2\times3H_2O$ (0.2 mmol, 48.3 mg dissolved in 3 mL DMF/EtOH (v/v, 2:1)) were mixed. And the resulting mixture was caped in a 20 mL Teflon lined steel autoclave and subjected to heat for 35 h at 90 °C in an air oven. After the reaction, around 100 mg of the solid powder obtained was filtered and washed with ethanol followed by vacuum drying at 50 °C overnight. The similar procedure was repeated separately by using 0.2 mmol of other metal nitrates *e.g.,* $Zn(NO_3)_2\times6H_2O$ (59.5 mg), $Co(NO_3)_2\times6H_2O$ (58.2 mg), and $Ni(NO_3)_2\times6H_2O$ (58.2 mg) to get their respective products. When the organic linker *5*-azidoisophthalic acid (0.2 mmol, 41.4 mg) was used a colloidal solution was obtained.

1.4. Synthesis of Cu-MOF

0.2 mmol of each of the 5-prop-2-ynoxyisophthalic acid (H_25-PIP, 44 mg) and 5-azidoisophthalic acid (H_25-N_3IP, 41.4 mg) and 0.5 mmol of $Cu(NO_3)_2\times2.5H_2O$ (116.3 mg) were dissolved in DMA:H_2O (4:1, 15 mL), then drops of H_3BO_4 were added into the mixture solution. The mixture was placed in a capped 20 mL vial and heated at 85 °C in an oven for 2 days. The blue block crystals of CuMOF suitable for X-ray diffraction were obtained after cooling down to room temperature. The crystals were washed by fresh DMA for three times and dried naturally at room temperature to yield Cu-MOF (100 mg) [Elemental analysis found (%) C 41.94, H 6.045, and N 9.88].

1.5. Synthesis of ligand (H_4L)

Sodium acetate (0.3 mmol, 59.4 mg) and $CuSO_4\times5H_2O$ (0.03 mmol, 7.5 mg) are added to solvent mixture of methanol, water and tertiary- butanol (1:1:1, v/v/v, 15 mL) containing 5-prop-2-ynoxyisophthalic acid (3 mmol, 660 mg) at room temperature under inter atmosphere i.e., nitrogen. 5-azidoisophthalic acid (3 mmol, 621.1 mg) is added to the previous solution and stirred overnight. White precipitate was filtered, washed with water, and dried under vacuum at 80 °C for 12 h to get 760 mg of ligand *i.e.*, 5-(4-((3,5-dicarboxyphenoxy)methyl)-1*H*-1,2,3-triazol-1-yl)isophthalic acid (H_4L). NMR spectra (500 MHz) for ^{1}H and ^{13}C in DMSO-d^6 are measure for δ_H (2H 5.41 (OCH_2-) s; 2H 7.82 (Ar-H) d; 1H 8.11 (Ar-H) t; 1H 8.52 (Ar-H) t; 2H 8.64 (Ar-H) d; 1H 9.25 (N3C2-H) s; and 4H 13.49 (COOH) br) ppm, and δ_C (167.05, 158.53, 144.39, 137.32, 133.74, 133.03, 130.12, 124.76, 123.74, 123.16, 120.12, 72.59) ppm, respectively. FTIR spectrum resulted in ν (3745, 3560, 3087, 3001, 2518, 1917, 1691, 1591, 1460, 1417, 1274, 1220, 1043, 1000, 927, 904, 835, 756, 709, 661, 609, 507, 459) cm^{-1}.

1.6. Synthesis of Zn-MOF

0.1 mmol of each of the H_4L (42.6 mg) and $Zn(NO_3)_2\times6H_2O$ (65 mg, 0.22 mmol) dissolved in *N,N'*-dimethylformamide (DMF, 12 mL). The solution was placed in a tightly capped 15 mL glass vial and heated at 75 °C in an oven for 3 days. The colorless needle-like crystals were collected. The crystals were washed by fresh DMF for 3 times and (15 mg) [Elemental analysis found (%): C 36.16, H 2.41, and N 6.70].

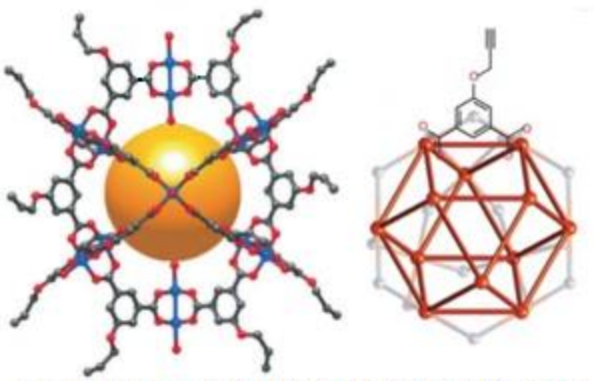

[Cu_{24}(5-prop-2-ynoxyisophthalate)$_{24}$(H_2O)$_{24}$] (CuMOP)

Figure S1. Presentation of the alkyne covered surface of the metal-organic polyhedron (CuMOP) in a molecular (**left**) and a cuboctahedral cage like fashion (**right**).[1]

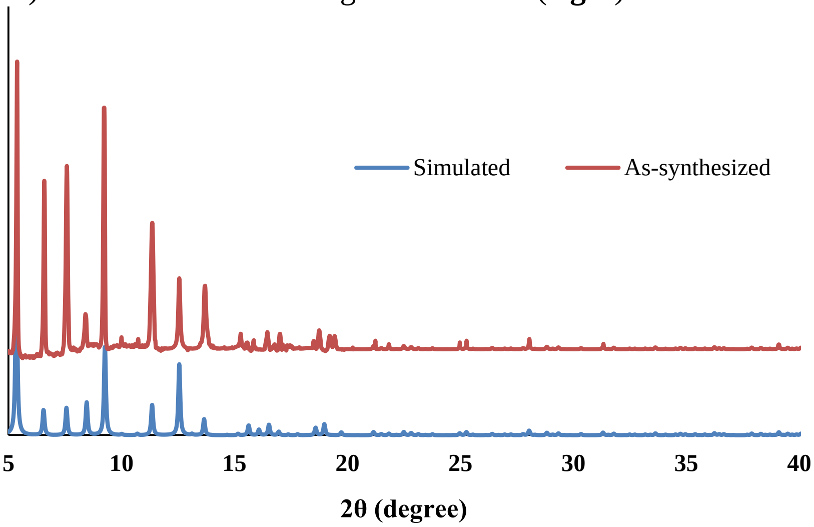

Figure S2. Powder XRD of the simulated and as-synthesized crystals of CuMOP.

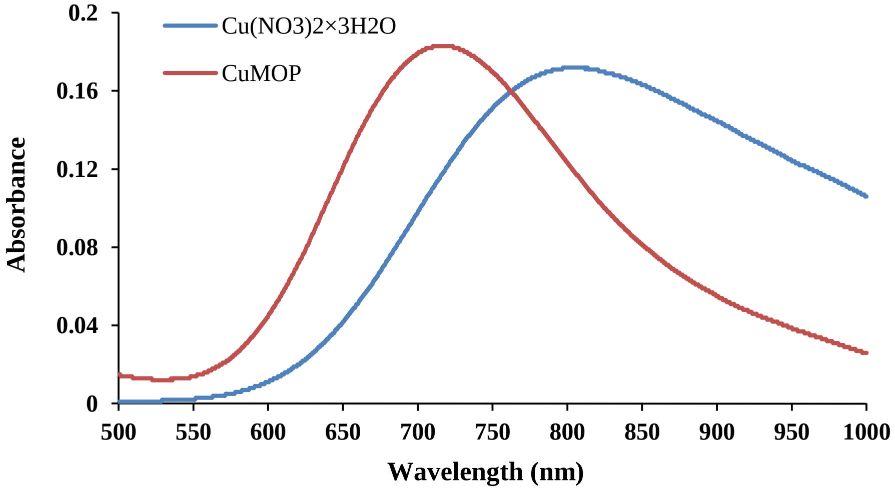

Figure S3. UV-Vis spectra of the of $Cu(NO_3)_2{\times}3H_2O$ and as synthesized CuMOP in DMF.

2. Stability of CuMOP

The UV/Vis spectroscopic measurements of CuMOP solution in DMF were recorded at different time interval to check its stability at room temperature. The peaks at 718 nm in DMF solution indicates the presence of Cu-Cu paddle-wheels in the MOP structure. And, it is found to be quite stable as shown in Fig. S4. The chemical stability of the CuMOP solution in DMF was checked in the presence of water, acid (HCl), and base (NaOH). 5 mg of the CuMOP was dissolved in 3 mL of the DMF solution followed by the addition of the 100 μL of water and the UV/Vis spectra were recorded at different time intervals (Fig. S5). Similarly, UV/Vis spectra were recorded after the addition of 100 μL of 0.1 M HCl and 0.1 M NaOH aqueous solutions (Fig. S6 and S7). CuMOP was found to be stable in water, whereas in the case of acidic and basic conditions the MOP structure collapsed as evidenced by the disappearance of the Cu-Cu paddle-wheel peek at 718 nm. Furthermore, the stability of the CuMOP was also checked under the reaction conditions (solvothermal heating DMF/EtOH (v/v, 2:1) at 90 °C for 35 h). The UV-Vis spectra (Fig. S8) indicted that Cu-Cu paddle-wheel peak (718 nm) was maintained and this suggested the presence of the same material *i.e.*, CuMOP in solution for both cases, otherwise for the free Cu^{2+} ions in the solution a peak should appear at 800 nm (as shown in the Fig. S3 UV-Vis spectrum of $Cu(NO_3)_2$ solution).

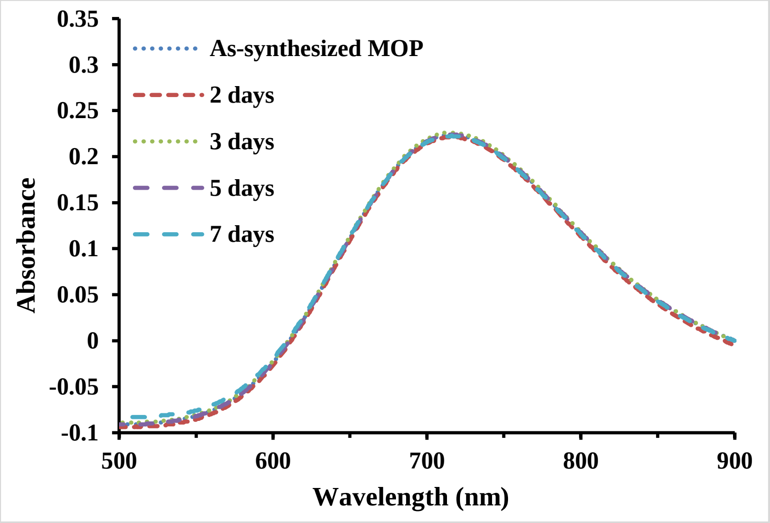

Figure S4. UV-Vis spectra at different time intervals showing CuMOP stability in DMF solution at room temperature

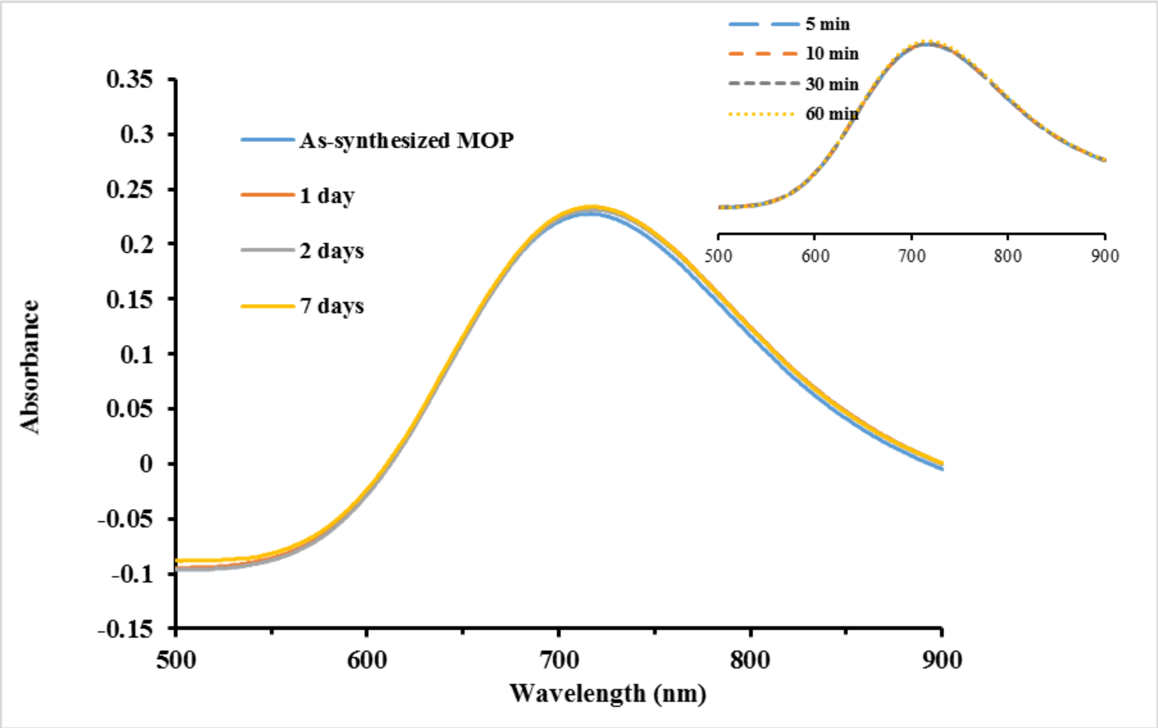

Figure S5. UV-Vis spectra at different time intervals showing CuMOP stability in DMF solution after adding 100 µL of water

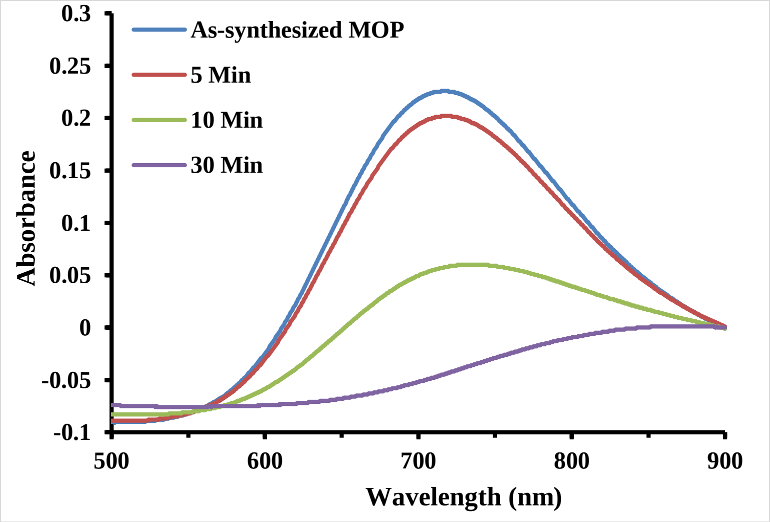

Figure S6. UV-Vis spectra at different time intervals showing CuMOP stability in DMF solution after adding 100 µL of 0.1 M HCl solution.

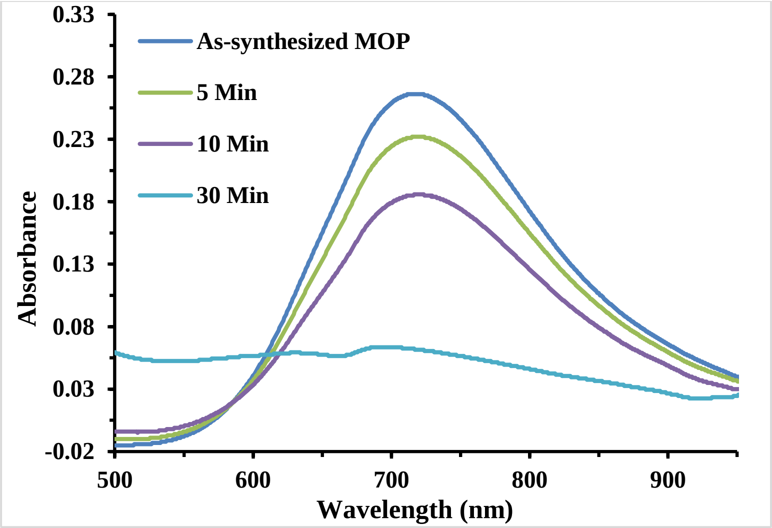

Figure S7. UV-Vis spectra at different time intervals showing CuMOP stability in DMF solution after adding 100 μL of 0.1 M NaOH solution.

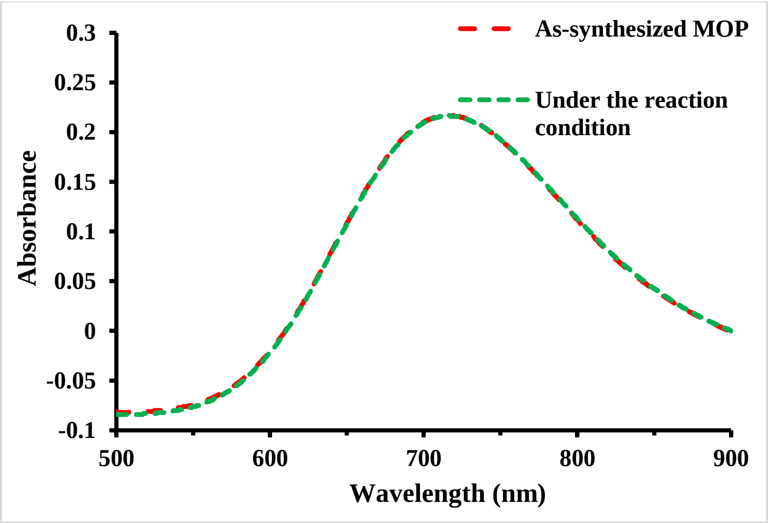

Figure S8. UV-Vis spectra of CuMOP in DMF/EtOH (v/v, 2:1) before and after the solvothermal heating at 90 °C for 35 h (reaction conditions).

3. NMR and ESI-MS spectra

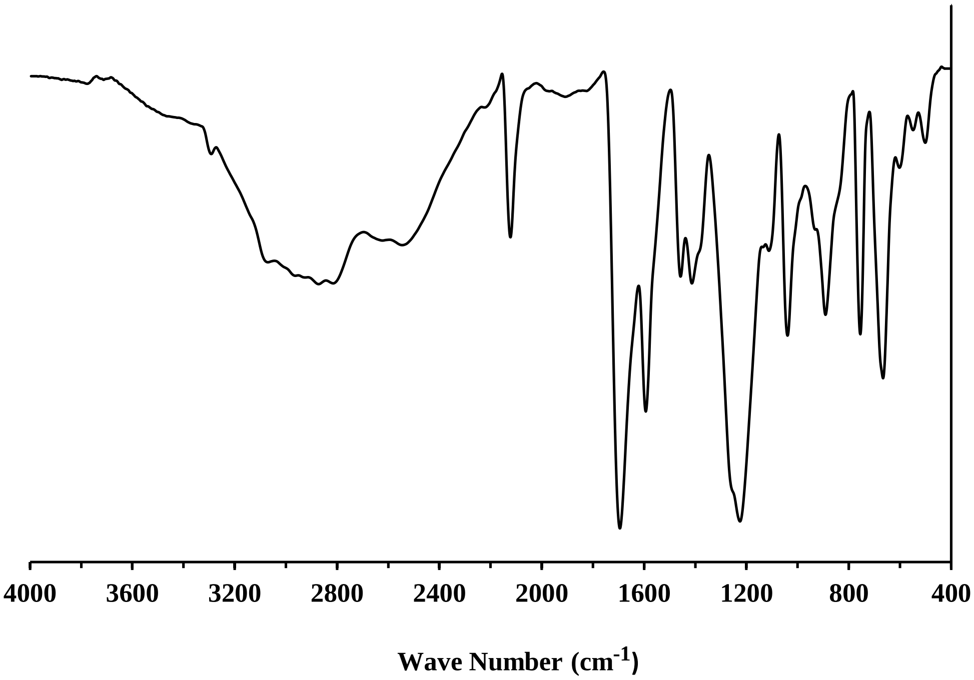

Figure S9. FTIR spectrum from the solvothermal reaction of 5-azidosophthalic acid (H_25-N3IP) with 5-prop-*2*-ynoxyisophthalic acid (H_25-PIP) in *N*, *N'*-dimethylformamide (DMF) at 90 °C for 24 h, as a control reaction.

In order to confirm the regioselective formation of 1*H*-1,2,3-triazole moiety in the ligands, the degradation of coordination polymeric networks was carried out. 40 mg of as-synthesized products were washed firstly with distilled MeOH and then stirred in 2 mL of 2N NaOH aqueous solution at room temperature. After six hours, the resulting black solids were separated by centrifugation to obtain a colorless solution. To this solution 1N HCl aqueous solution was added until pH = 3 and white precipitate formed. The water was removed from the system under vacuum to give a white solid, to which DMSO-d^6 was added for the measurement of the ^{1}H NMR.

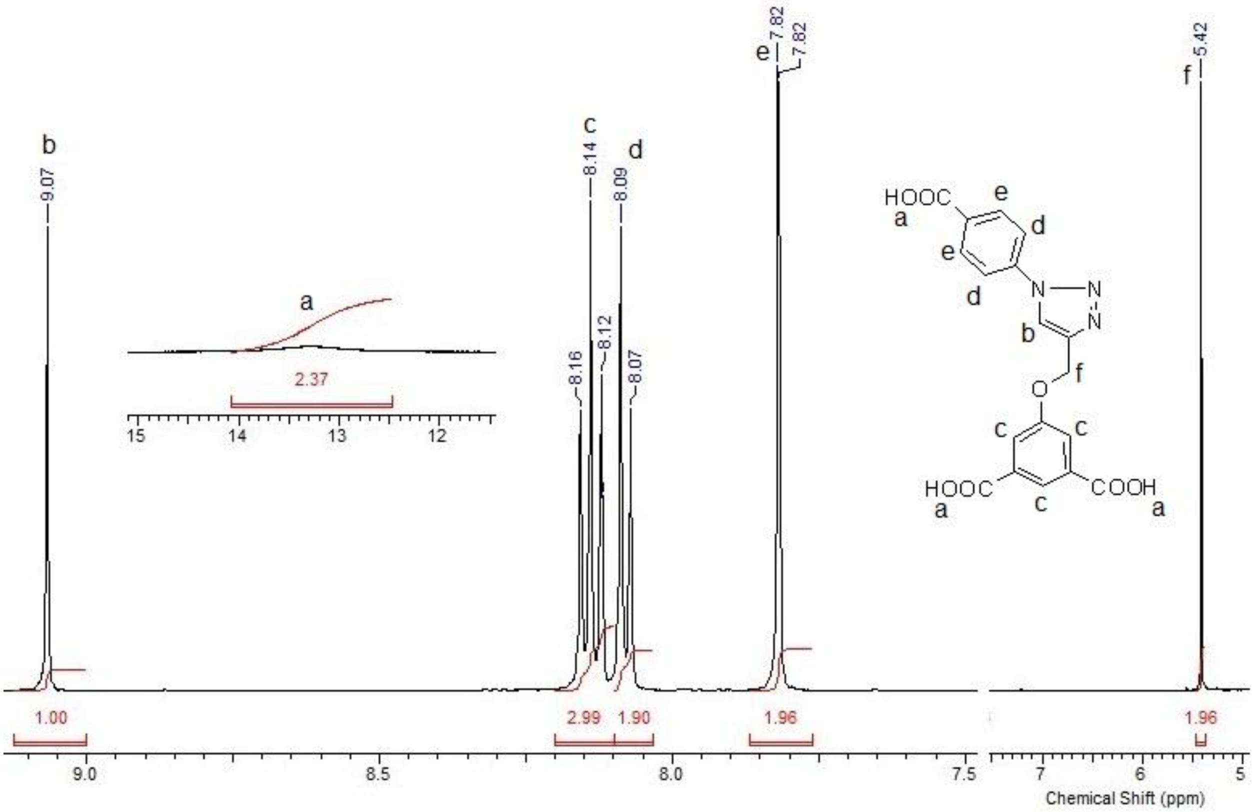

Figure S10. ^{1}H-NMR spectrum of the ligand 5-((1-(4-carboxyphenyl)-1*H*-1,2,3-triazol-4-yl)methoxy) isophthalic acid obtained after the degradation process of the CuMOP-N1(Cu).

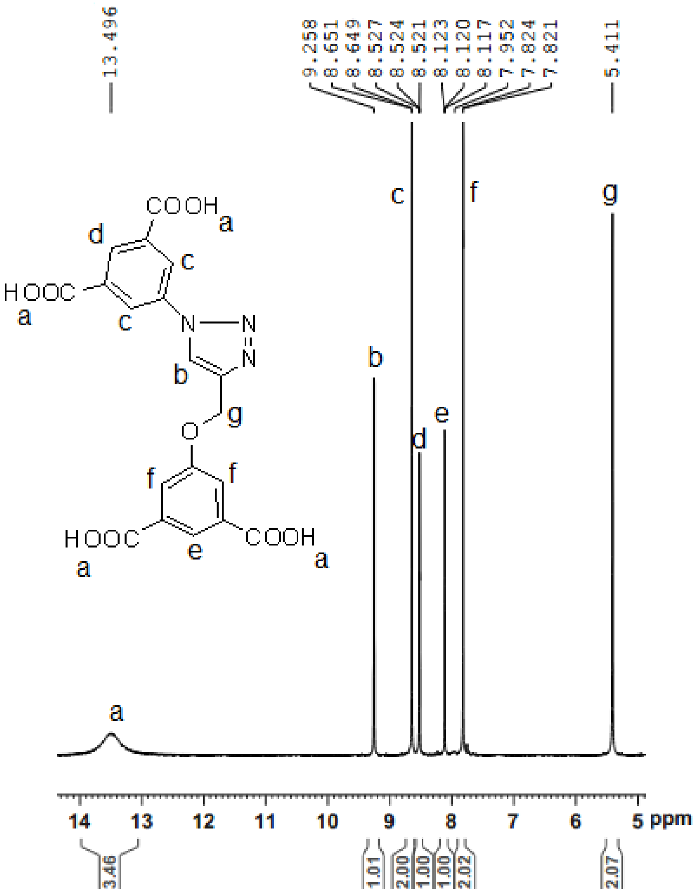

Figure S12. ^{1}H-NMR spectrum of the ligand 5-(4-((3,5-dicarboxyphenoxy)methyl)-1*H*-1,2,3-triazol-1-yl) isophthalic acid isolated after the degradation process of the CuMOP-N2(Cu).

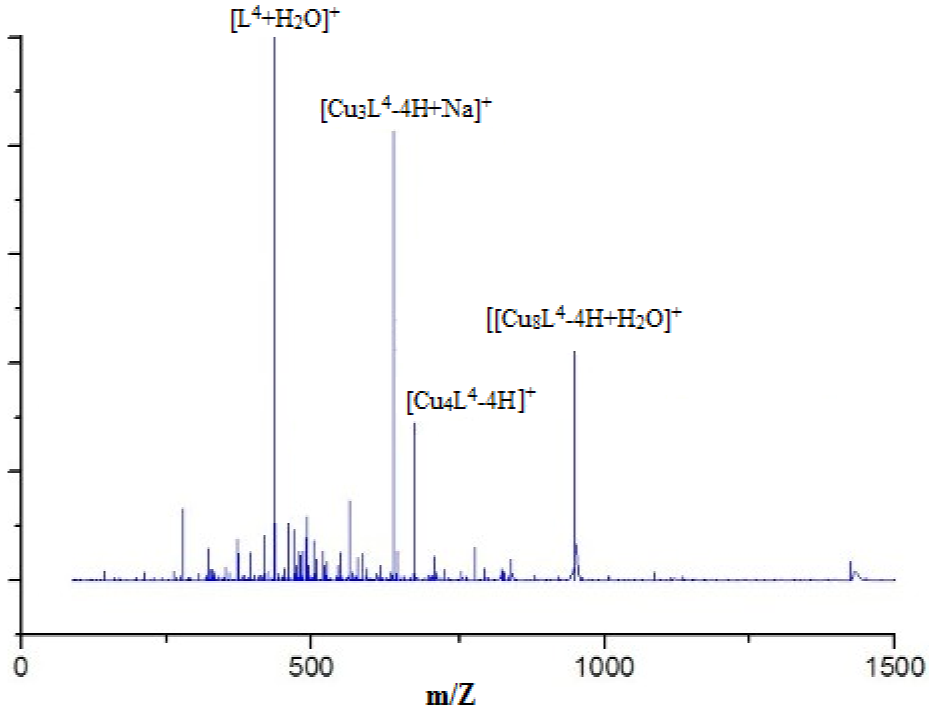

Figure S12. Electrospray mass spectrum (ESI-MS) of the CuMOP-N2(Cu) obtained from the supernatant after centrifuging the 0.5 mL CuMOP-N2(Cu) with 9.5 mL methanol. L is the ligand 5-(4-((3,5-dicarboxyphenoxy)methyl)-1*H*-1,2,3-triazol-1-yl)isophthalic acid.

4. CHN analysis

According to the CHN analysis, the found values are very close to the calculated values. The calculations of the CHN contents are based on the fact that CuMOPs remain intact within the overall polymeric structure of the network (Table S1). The metallic contents other than copper (which is contributed by CuMOP) for networking are calculated assuming that 12 sites around the MOP create network through clicked linkers N1 and N2 *i.e.*, *p*-azidobenzoate and 5-azidoisphthalate, respectively. Solvent molecules (DMF and water) are present as part of the bulk structure in the coordinated form to metal centers or having weak interactions, and/or as encapsulated in the cavities.

Table S1. CHN analysis of the CuMOP-N(M)s are described by comparison of the calculated and found values.

**Polymer Sample**	**C** (%) Found(calcd)	**H** (%) Found(calcd)	**N** (%) Found(calcd)
CuMOP-N1(Cu)	42.00(40.92)	3.70(3.24)	8.83(8.84)
CuMOP-N1(Zn)	41.44(40.79)	3.71(3.23)	8.75(8.81)
CuMOP-N1(Co)	40.64(40.20)	3.85(3.78)	8.39(8.41)
CuMOP-N1(Ni)	40.43(40.21)	4.03(3.78)	8.21(8.42)
CuMOP-N2(Cu)	38.75(38.90)	4.08(3.46)	8.85(8.25)

5. ICP analysis

The percentages of the total metal contents in the overall polymeric network are found by the inductively coupled plasma (ICP). The observed measurements of the metal contents (wt. %) in the networking are compared with the theoretically calculated values. For the CuMOP-N1/N2(M)s, the samples were prepared by using Cu(II), Zn(II), Co(II) and Ni(II)-salts along with networking linkers N1/N2, respectively, resulting in the overall metal contents as listed in the following table S2. The observed weight percentage (wt. %) of the copper matches very well with the theoretically calculated values ensuring that Cu(II) contents of the CuMOP in their polymeric network were not affected. The description of the metal motivating the coordination driven self-assembling of the CuMOP is interesting to follow.

Table S2. ICP analysis of the CuMOP-N1(M) are described by the comparison of the calculated and found values.

**Sample**	**Copper(II)** / wt.% Found(calcd) [a, b]	**M(II)** / wt.% Found(calcd) [a, b]
CuMOP-N1(Cu)	23.49(23.57)	-
CuMOP-N1(Zn)	09.57(11.77)	Zn: 11.87(12.11)
CuMOP-N1(Co)	11.08(12.60)	Co: 06.12(05.84)
CuMOP-N1(Ni)	12.47(12.61)	Ni: 04.42(05.82)
CuMOP-N2(Cu)	21.31(21.81)	-

[a] Calculated values are based on the assumptions that CuMOPs remain intact within the overall polymeric structure of the network. [b] The metallic contents other than copper (contributed by CuMOP) for networking are calculated assuming that 12 sites around the MOP create network through clicked linkers N1 and N2 *i.e. p*-azidobenzoate and 5-azidoisphthalate, respectively.

This reveals that out of 24 carboxylic sites around the CuMOP (generated after the click of the terminal 24 alkyne functional groups with the H *p*-N_3B), only half them were coordinated and created a coordination driven self-assembling among them leaving the other half as free carboxylic sites. The stereochemical requirements of the coordination driven self-assembling of the MOP cages with each other make it quite understandable that all of the 24 coordination sites created by the click of the *p*-azidobenzoic acid cannot be entertained. Hence, the calculated and found values agree with each other very well. The same was found in the case of Zn(II) as mentioned in table S2. Unlike Cu(II) and Zn(II), the Co(II) and Ni(II) are different because these do not involve paddlewheel SBUs instead coordinates with two carboxylate moieties. Therefore, these would have mononuclear coordinate with the carboxylate moieties thereby giving low metallic wt. % contents than the former case. Therefore, Co and Ni accounts for the twelve (12) ions (*i.e.,* ~5.8%) around one cage unit on the whole for networking leading to the polymeric structure.

6. Magnetic measurements:

The cooperative magnetism in $Cu_2(COO)_4$ binuclear paddlewheel is acknowledged to primarily exhibit the antiferromagnetic coupling. The copper based organic-inorganic structures have been associated with the combined intradimer and interdimer magnetic interactions. The involvement of the other metal centers like cobalt, nickel or zinc affects magnetic properties of the framework. The magnetic properties of CuMOP-N1(M(II)) are featured in following figures. The temperature-dependent magnetic moments at a constant field (1000 Os) is shown in Figure S8 and the field-dependent magnetization at low temperature (5 K) in Figure S9.

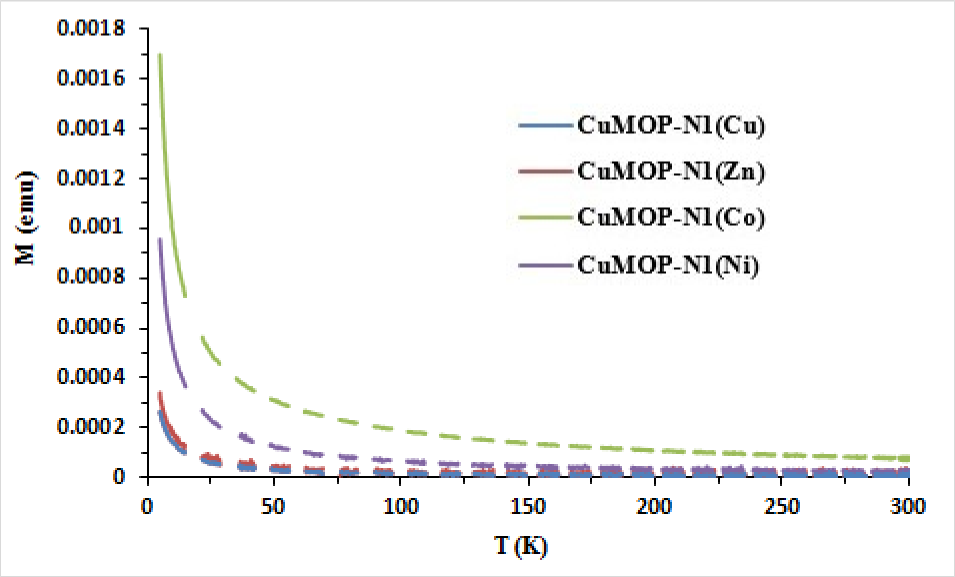

Figure S13. The temperature-dependent magnetic moment (emu) at 1000 Os (data points) for the CuMOP-N(M).

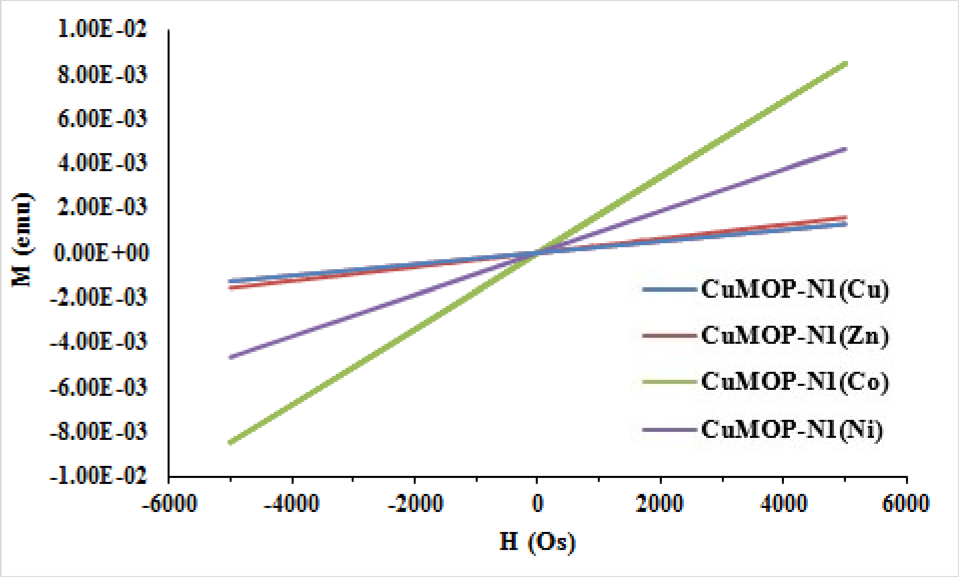

Figure S14. The field-dependent magnetization (emu) at 5 K (data points) for the CuMOP-N(M).

7. SEM, EDS, TEM, PXRD, and TGA analysis

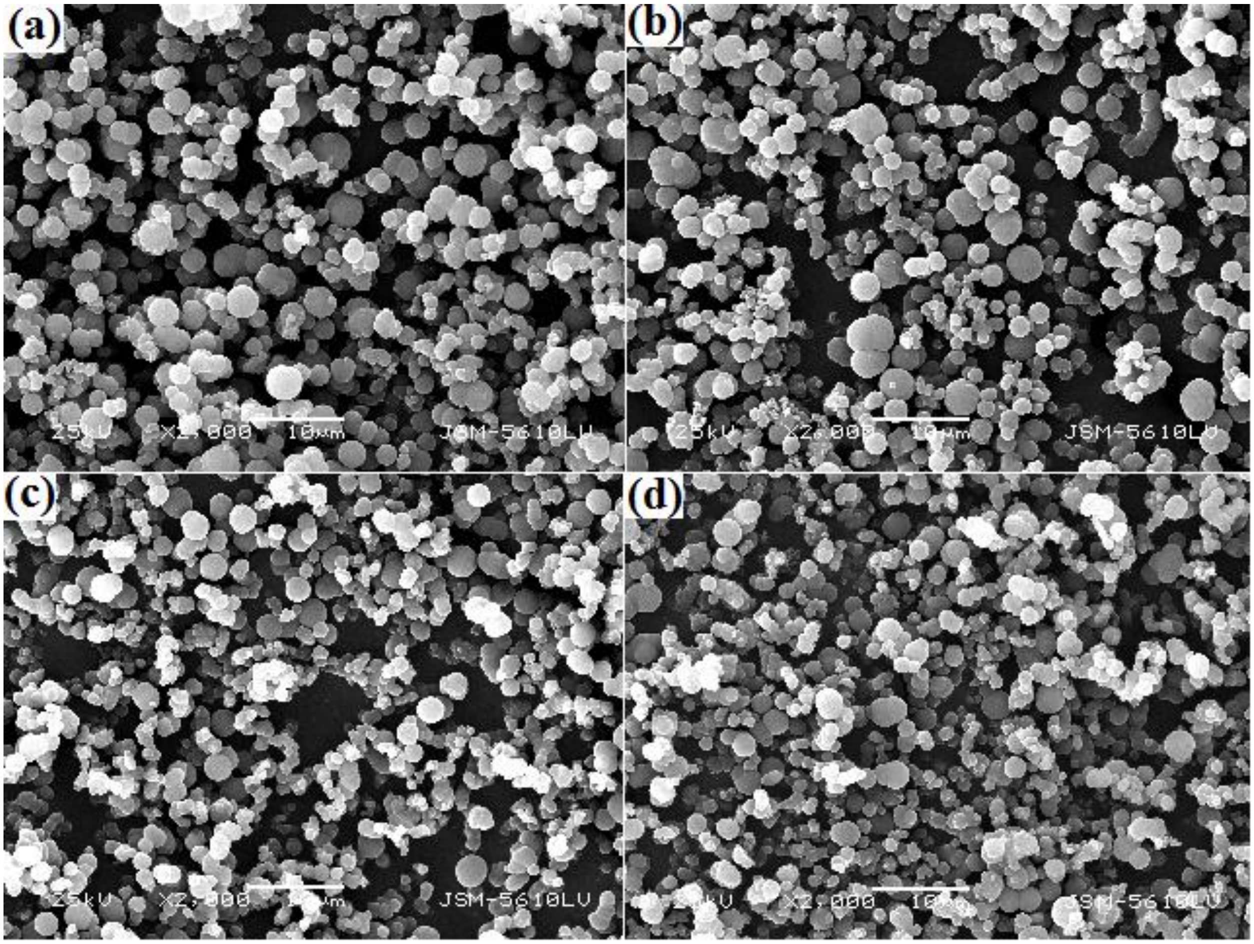

Figure S15. SEM images of the CuMOP-N1(M) series: (a) CuMOP-N1(Cu), (b) CuMOP-N1(Zn), (c) CuMOP-N1(Co), and (d) CuMOP-N1(Ni).

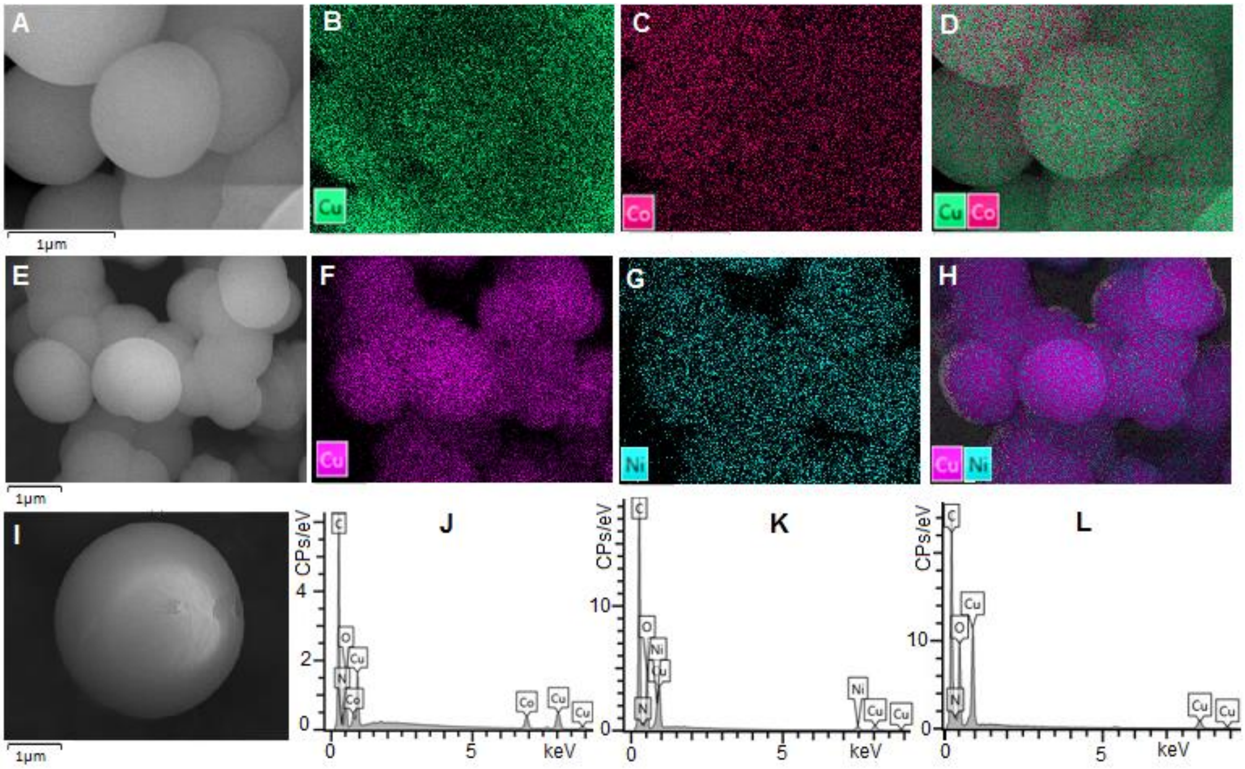

Figure S16. FE-SEM images and EDS metal mapping analysis of the CuMOP-N1(Co) (**A**-**D**), CuMOP-N1(Ni) (**E**-**H**), CuMOP-N1(Cu). EDS compositional analysis of the CuMOP-N1(Co). (**J**) CuMOP-N1(Ni) (**K**), and CuMOP-N1(Cu) (**L**) obtained from overall elemental mapping analysis.

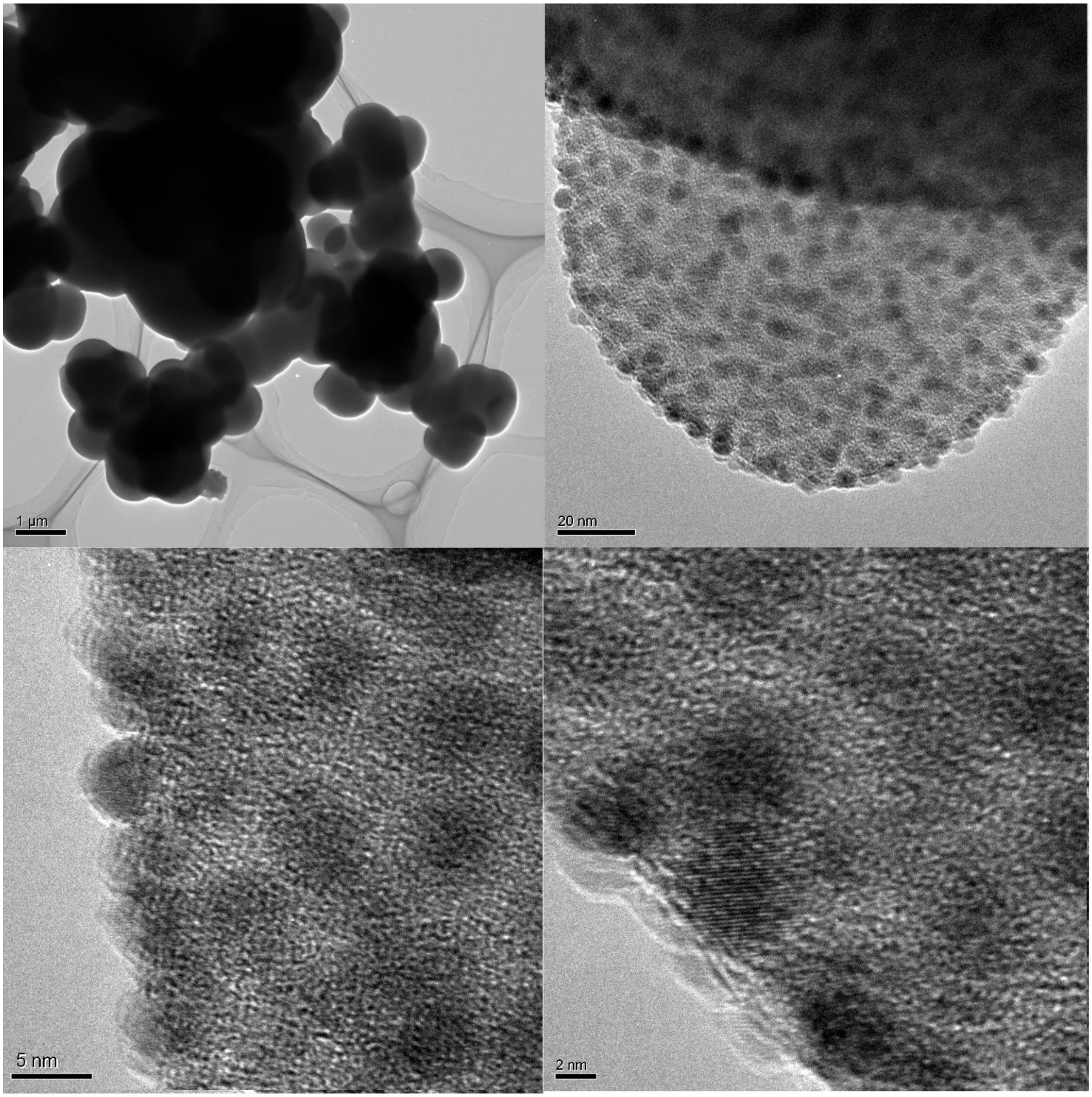

Figure S17. TEM analysis of the CuMOP-N1(Ni).

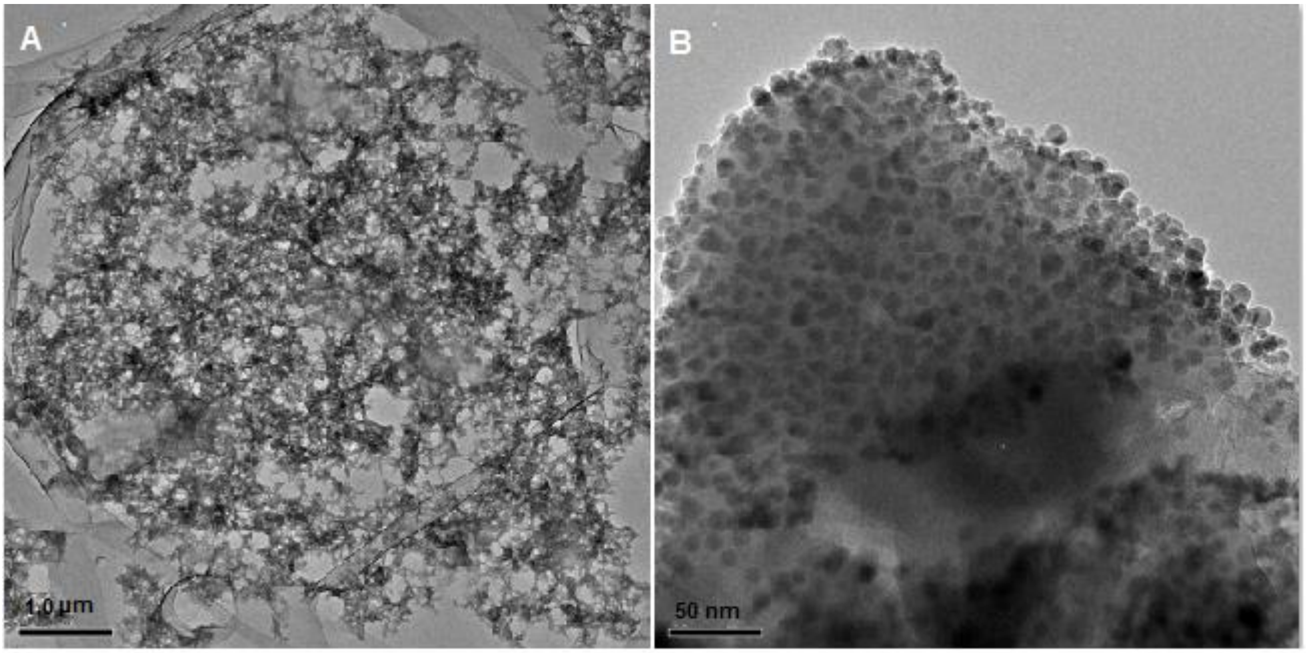

Figure S18. TEM analysis of the CuMOP-N2(Cu).

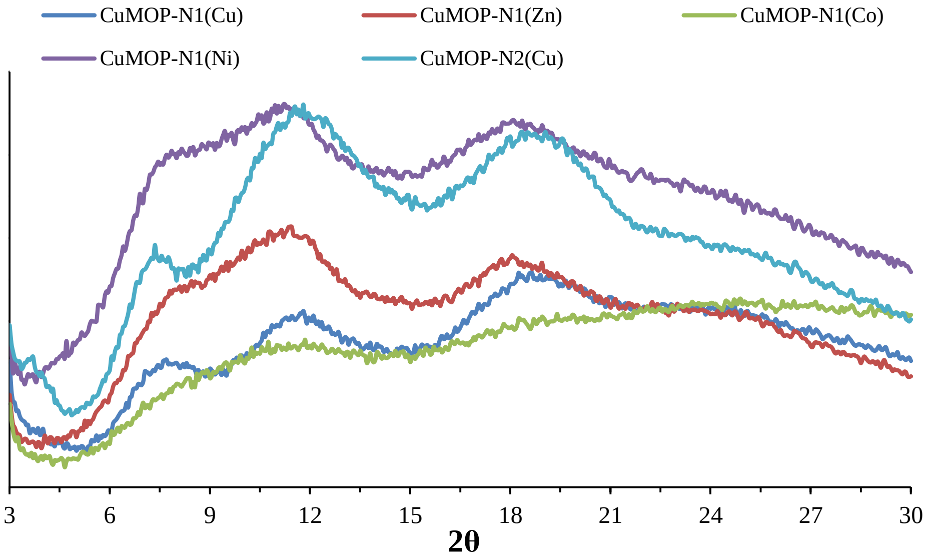

Figure S19. Powder XRD spectra for CuMOP-N(M).

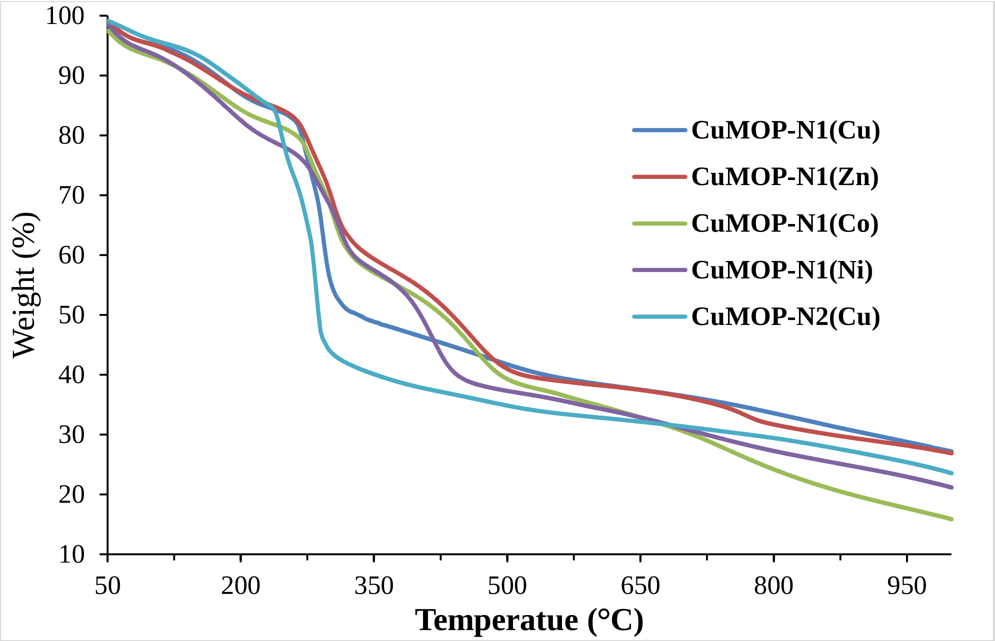

Figure S20. TG plots for CuMOP-N(M).

8. Single crystal X-ray diffraction

For the structure of compound Cu-MOF, X-ray intensity data were collected on a Agilent Supernova Dual Source (Cu at zero) diffractometer equipped with an Atlas CCD detector using CuKα radiation (λ = 1.54178 Å) and ω scans. For the structure of compound Zn-MOF, X-ray intensity data were collected on a Bruker diffractometer equipped with an APEX II CCD detector using MoKα radiation (λ = 0.71073 Å) and φ and ω scans. For Cu-MOF, the images were interpreted and integrated with the program CrysAlisPro (Agilent Technologies). For Zn-MOF, the images were interpreted and integrated with the program SAINT from Bruker. Using Olex2,[3] the structures were solved by direct methods using the ShelXS structure solution program and refined by full-matrix least-squares on F^2 using the ShelXL program package.[4] For both structures, non-hydrogen atoms were anisotropically refined and the hydrogen atoms in the riding mode and isotropic temperature factors fixed at 1.2 times U(eq) of the parent atoms. The contribution of heavily disordered solvent molecules was taken into account and suppressed using the SQUEEZE procedure in PLATON.[5] CCDC-1418777 and CCDC-1418778 contain the supplementary crystallographic data for Cu-MOF and Zn-MOF respectively.

8.1 Crystal data for crystal structures and refinement details

Cu-MOF: $C_{19}H_9Cu_2N_3O_{11}$, M = 582.39, trigonal, space group R-3m (No. 166), $a = b$ = 18.7403(7) Å, c = 41.2715(17) Å, α = β = 90° γ = 120°, V = 12552.6(12) Å^3, Z = 9, T = 100 K, ρ_{calc} = 0.693 g cm^{-3}, μ(Cu-Kα) = 1.184 mm^{-1}, F(000) = 2610, 10558 reflections measured, 3080 unique (R_{int} = 0.0415) which were used in all calculations. The final $R1$ was 0.0758 ($I > 2\sigma(I)$) and $wR2$ was 0.2216 (all data).

Zn-MOF: $C_{28}H_{30}N_6O_{12}Zn_2$, M = 773.36, monoclinic, space group $P2_1$ (No. 4), a = 10.058(2) Å, b = 16.600(4) Å, c = 14.739(3) Å, α = γ = 90°, β = 94.750(4)°, V = 2452.4(9) Å^3, Z = 2, T = 100 K, ρ_{calc} = 1.047 g cm^{-3}, μ(Cu-Kα) = 1.025 mm^{-1}, F(000) = 792, 16008 reflections measured, 7982

unique (R_{int} = 0.0370) which were used in all calculations. The final $R1$ was 0.0491 ($I > 2\sigma(I)$) and $wR2$ was 0.1375 (all data).

8.2. Topological analysis

The analysis was performed with the ToposPro program package and the TTD collection of periodic network topologies [6]. The RCSR three-letter codes [7] were used to designate the network topologies.

8.2.1. Cu-MOF

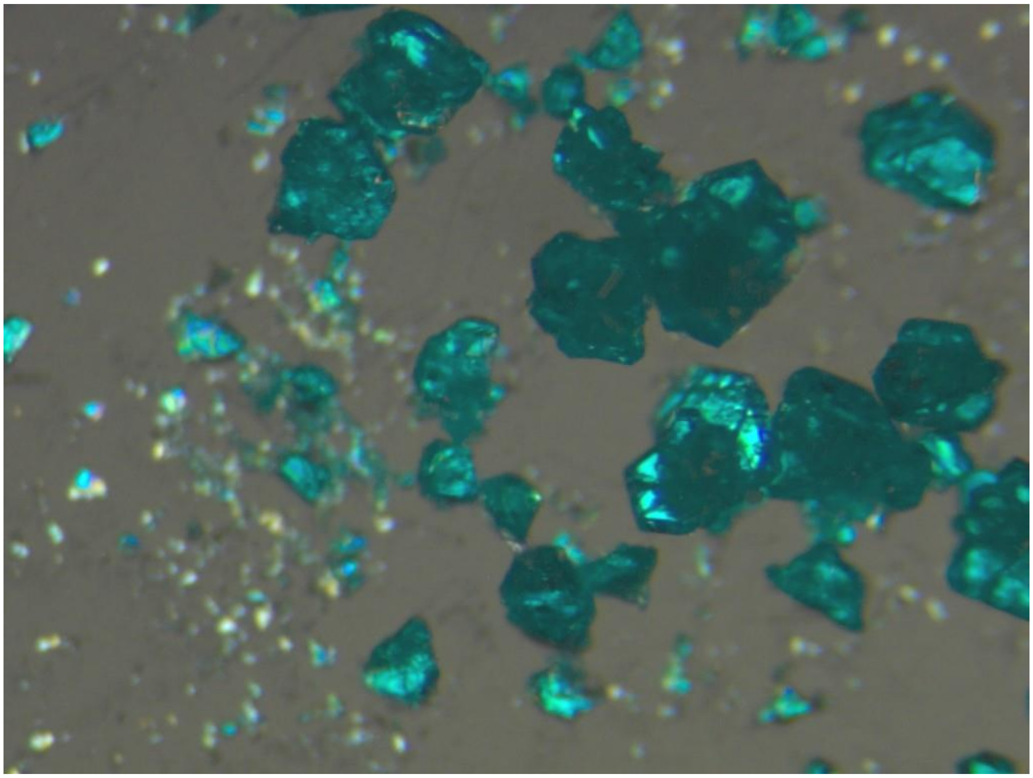

Figure S21. Microscopic picture of the as-synthesized block blue crystals of Cu-MOF suitable for single crystal X-ray diffraction analysis.

1) The compound is a metal-organic framework (MOF), consisting of Cu(II) ions and the triazole ligand "5-{4-[(3,5-dicarboxyphenoxy)methyl]-1*H*-1,2,3-triazol-1-yl}benzene-1,3-dicarboxylate".
2) The ligands are partially disordered (fig. S22); after lowering the crystal symmetry from *R*-3m to *R*3 and removing the disordered atoms the total composition is $C_{19}Cu_2H_9N_3O_{11}$.

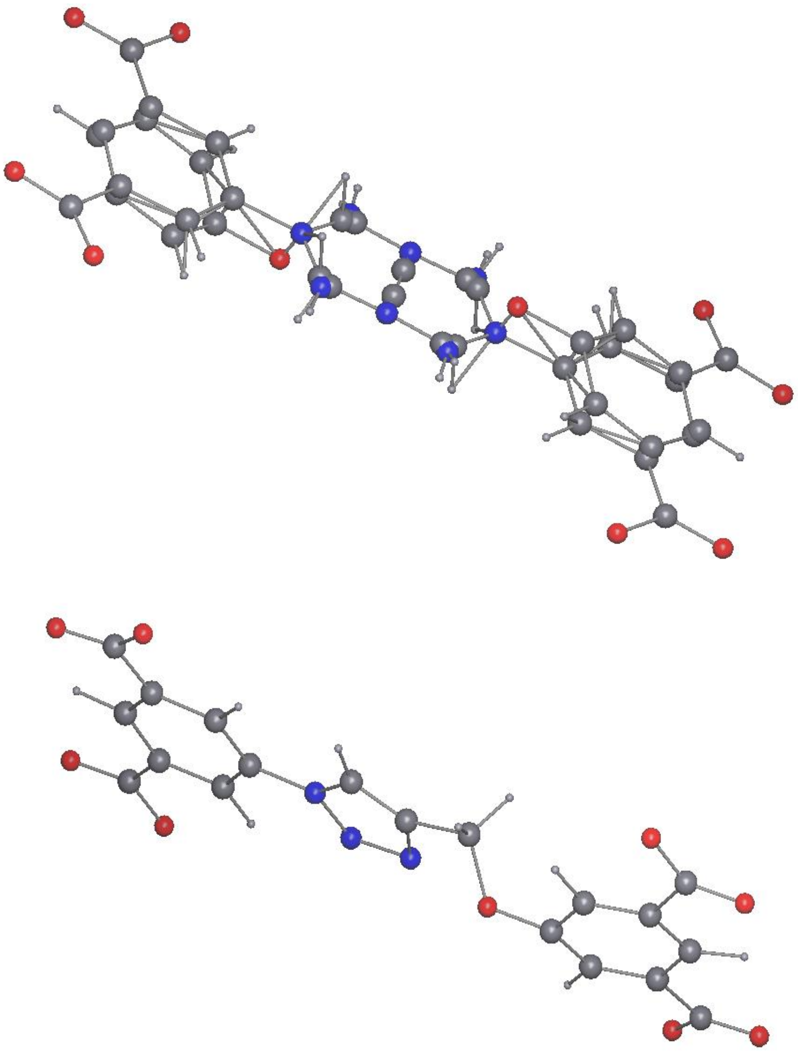

Figure S22. The triazole ligand "5-{4-[(3,5-dicarboxyphenoxy)methyl]-1*H*-1,2,3-triazol-1-yl}benzene-1,3-dicarboxylate", which is disordered over a mirror plane and a 2-fold axis (top) and the triazole ligand in the structure after removing the disorder (bottom).

3) The carboxylate groups of the ligands and the Cu(II) ions form paddle-wheel dimers; thus the resulting framework (fig. S22) can be simplified to its underlying net in two ways, the so-called standard and cluster representations [8].

(i) Standard representation

In the standard representation, the nodes of the underlying net are copper atoms and centers of mass of the organic ligands (fig. S23); the net topology after removing 0-, 1- and 2-coordinated nodes and ignoring the Cu-Cu contacts inside the dimer is found 4,8-coordinated 4,8T24, point symbol for the net: $\{4^4.6^{16}.8^8\}\{4^6\}_2$ (fig. S24).

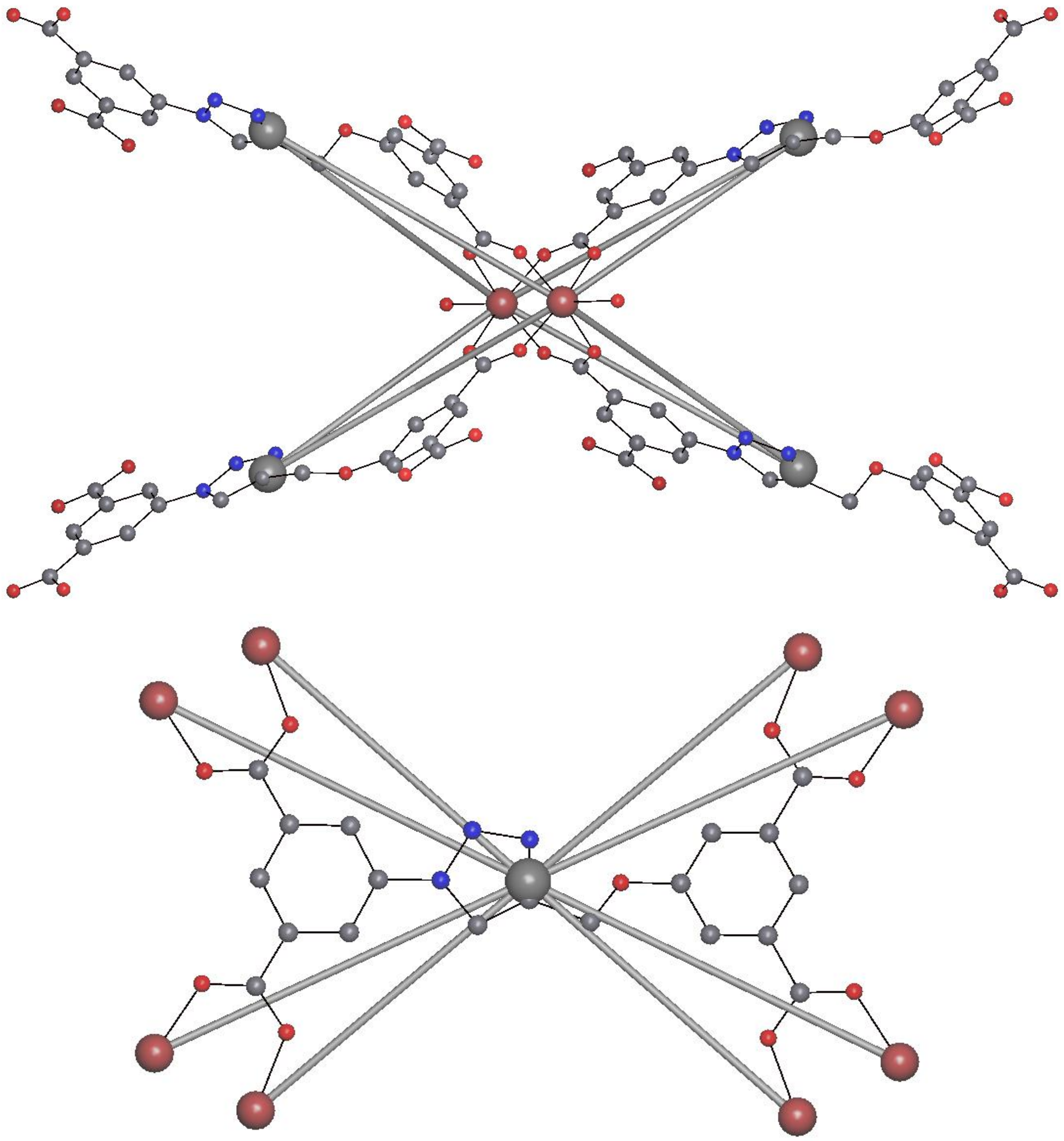

Figure S23. Nodes of the 4,8-coordinated underlying net in the standard representation: 4-coordinated Cu node (top) and 8-coordinated ligand node (bottom). The big grey balls coincide with the centers of mass of the ligands.

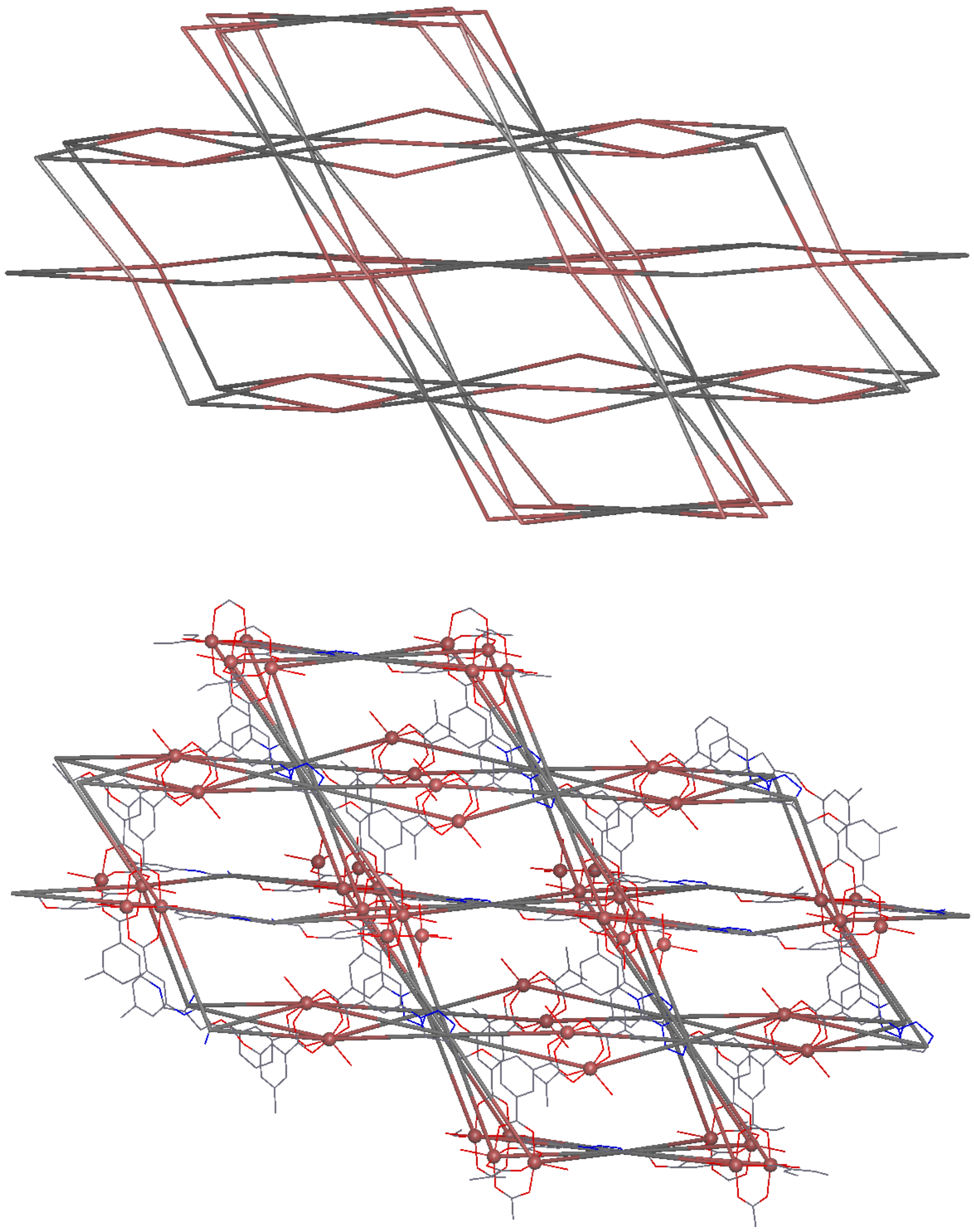

Figure S24. The underlying net 4,8T24 in the standard representation (top) and the net merged with the corresponding fragment of the framework (bottom).

(ii) Cluster representation

The cluster representation of the framework consists of 3-coordinated nodes corresponding to benzene rings of the ligand and 4-coordinated paddle-wheel dimer nodes (fig. S25). The underlying net of the framework in the cluster representations is 3,4-coordinated with a **fof** (*sqc*1575) topology (fig. S26).

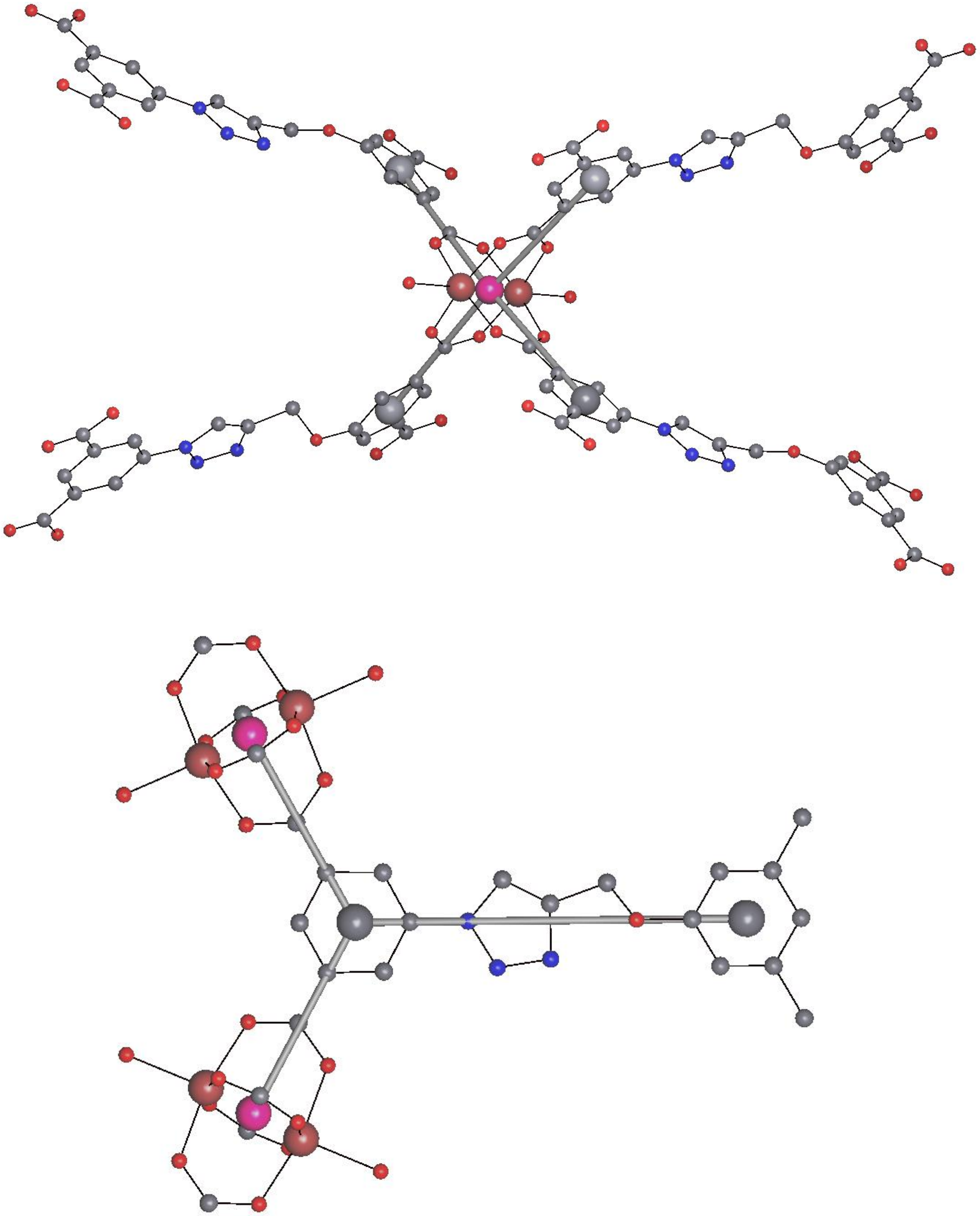

Figure S25. 4-coordinated paddle-wheel dimer nodes (pink balls) and 3-coordinated nodes (big grey balls) in the cluster representation.

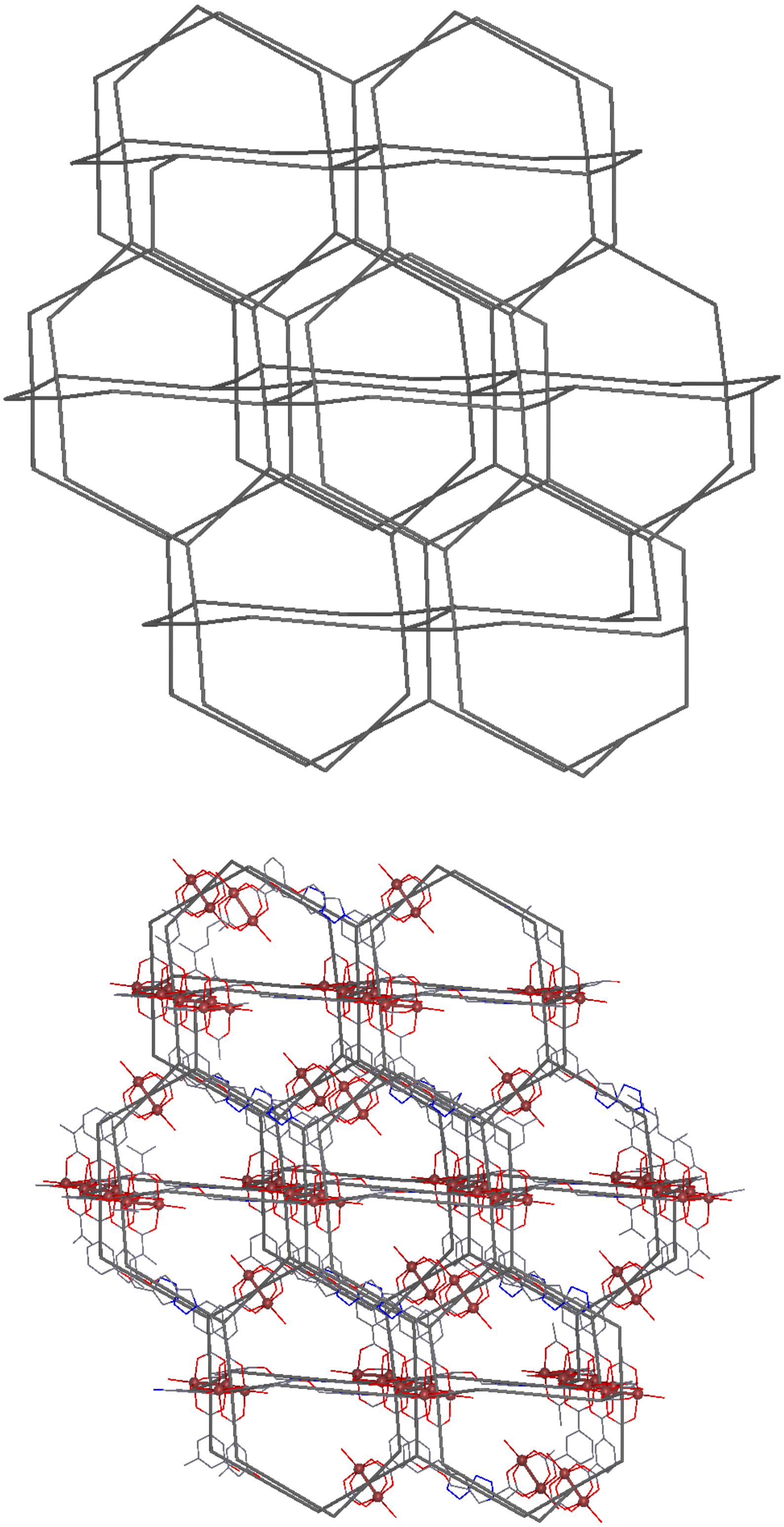

Figure S26. The underlying net in the cluster representation with a **fof** topology (top) and the net merged with the corresponding fragment of the framework (bottom).

8.2.2. Zn-MOF

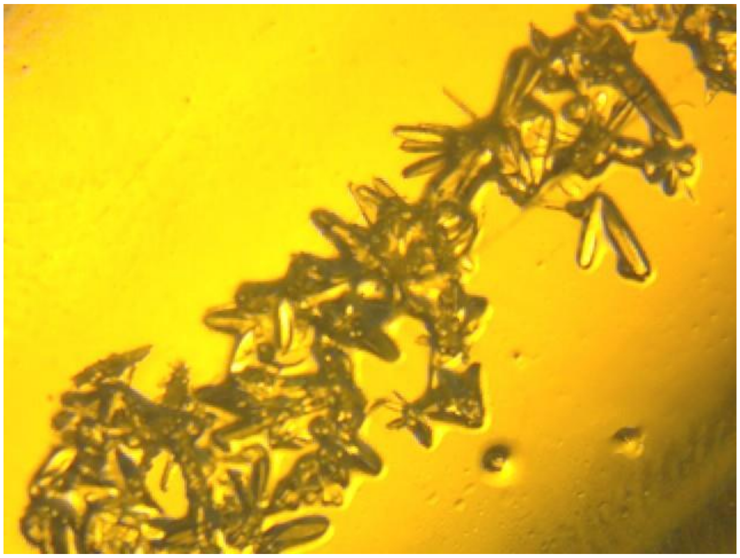

Figure S27. Microscopic picture of the as-synthesized needle-like crystals of Zn-MOF in mother liquor suitable for single crystal X-ray diffraction analysis.

The compound is a metal-organic framework (MOF), consisting of Zn(II) ions, polydentate ligands L = "5-{4-[(3,5-dicarboxyphenoxy)methyl]-1*H*-1,2,3-triazol-5-methyl-1-yl}benzene-1,3-dicarboxylate" and terminal DMF ligands. The total composition is $Zn_2(DMF)_3L$, $C_{28}H_{30}N_6O_{12}Zn_2$. The ligands L are partially disordered (fig. S28) and the metals form an asymmetric dimer (fig. S29).

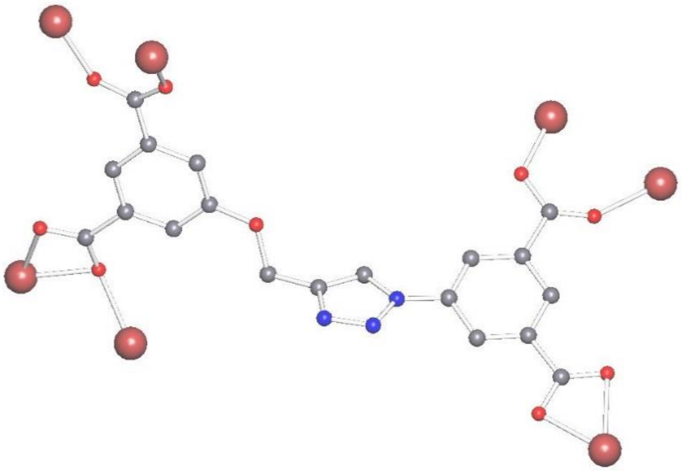

Figure S28. The triazole ligand L "5-{4-[(3,5-dicarboxyphenoxy)methyl]-1H-1,2,3-triazol-5-methyl-1-yl}benzene-1,3-dicarboxylate" in the Zn-MOF.

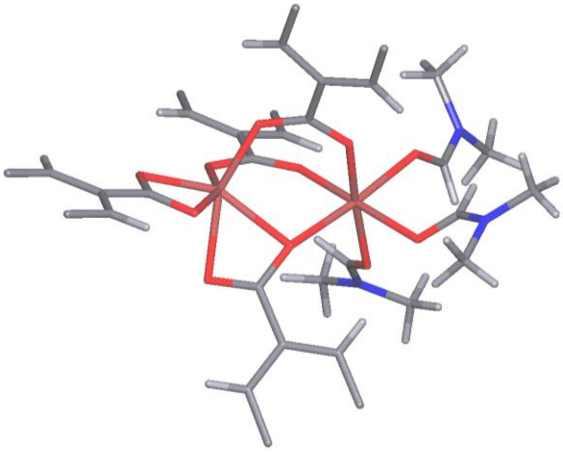

Figure S29. The carboxylate groups of four ligands, two Zn(II) ions and three terminal DMF form an asymmetric dimer (-COO)Zn(μ-COO)$_3$Zn(DMF)$_3$.

The resulting framework (fig. S30) can be simplified to its underlying net in two ways, the so-called standard and cluster representations [8].

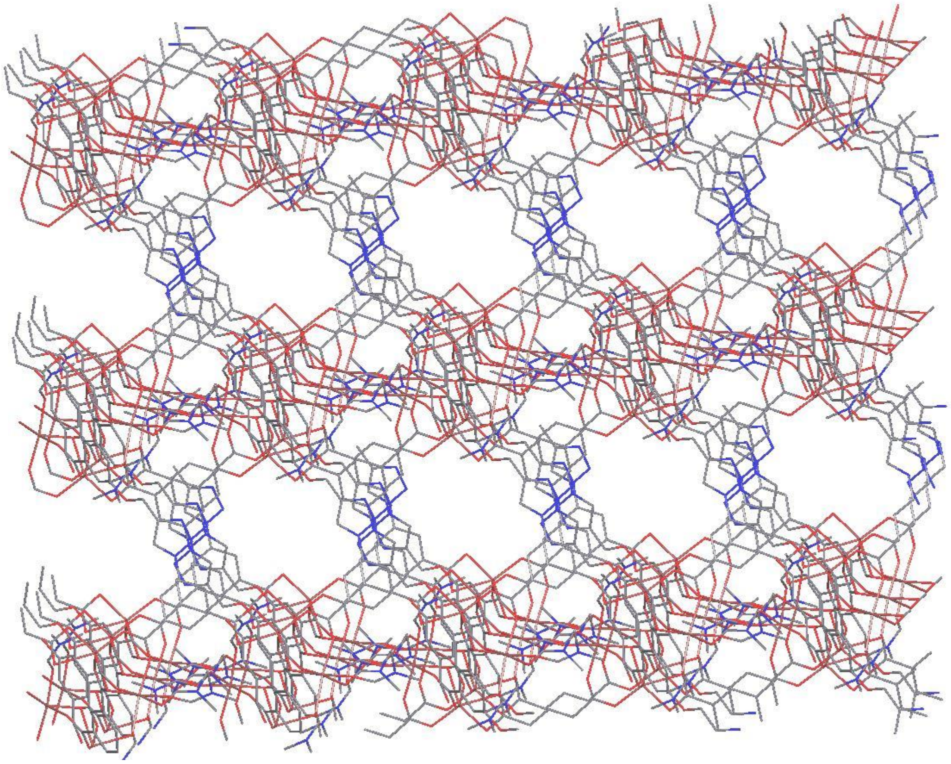

Figure S30. The framework of the structure before simplification.

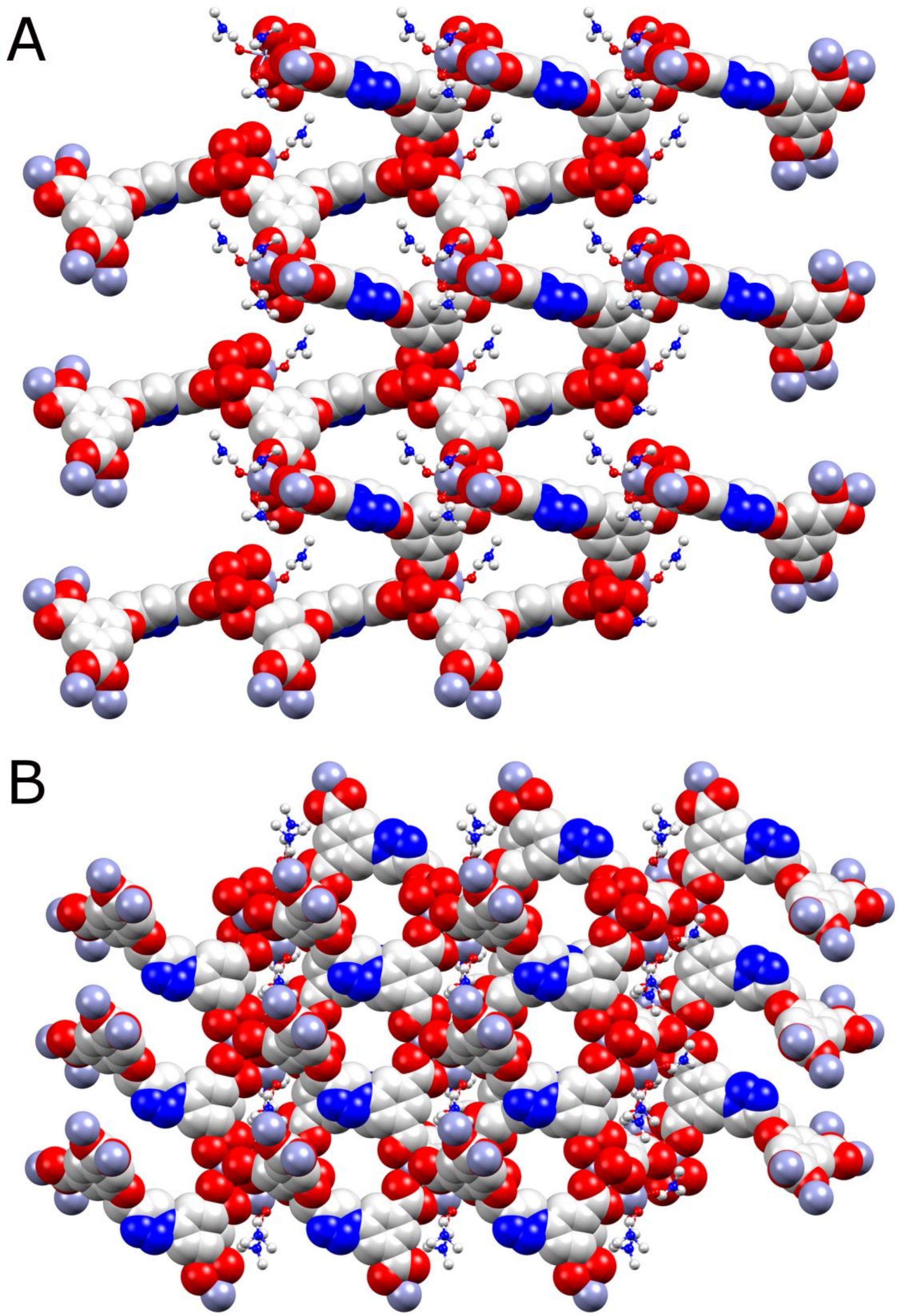

Figure S31. Packing diagram of the structure of compound Zn-MOF, showing the 2D grid-like solvent channels, built up by irregular solvent channels along the [100] direction (A), and smaller

elliptical cylindrical channels along the [010] direction (B). The framework atoms are depicted in space-filling (VDW radii) representation. The DMF molecules are depicted in ball-and-stick representation. Hydrogen atoms are omitted for clarity.

(i) Standard representation.

All zinc atoms and the centers of mass of the organic ligands are nodes of the underlying net in the standard representation (fig. S32). If we ignore the Zn-Zn contacts inside the dimer and remove 0-, 1- and 2-coordinated nodes, then the net topology is found 3,4,7- coordinated 3,4,7T57 (fig. S33).

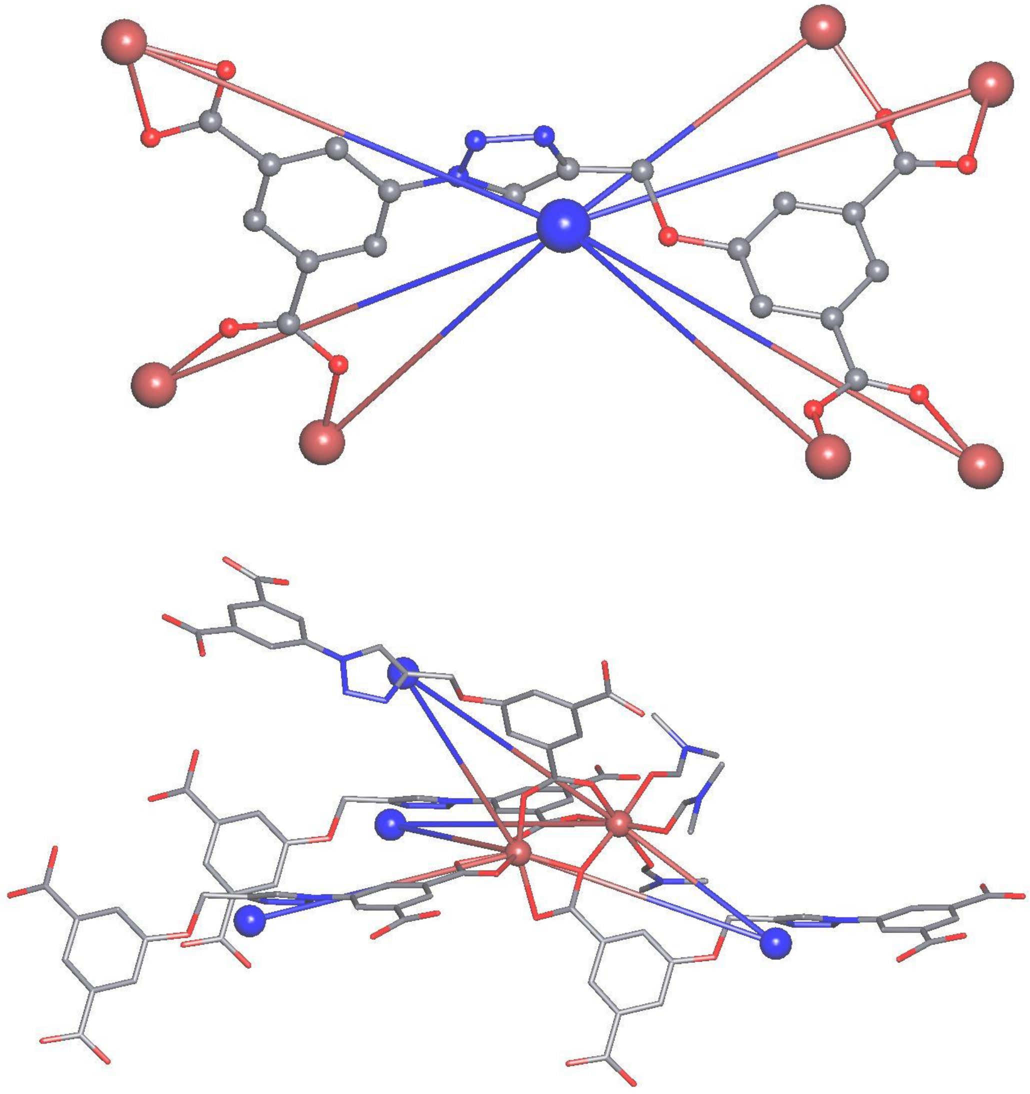

Figure S32. A 7-coordinated ligand in the standard representation. The big blue ball coincides with the centers of mass of the ligands (top). The asymmetric dimer gives two different Zn nodes, 4- and 3-coordinated. The underlying net it is then 3,4,7-coordinated (bottom).

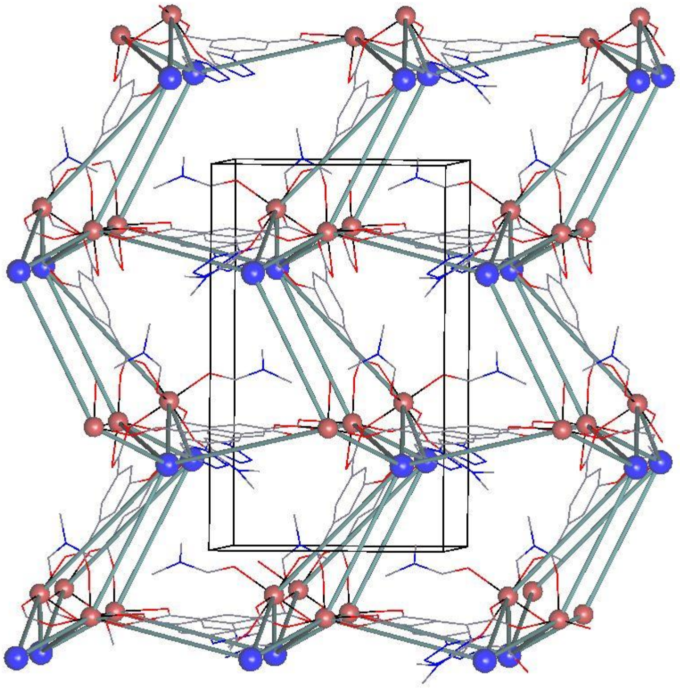

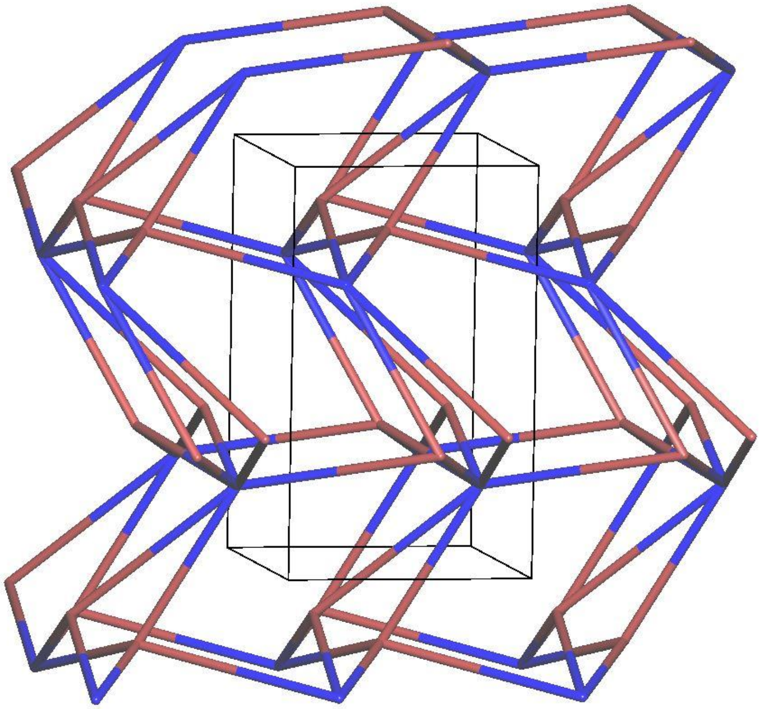

Figure S33. Two views of the underlying net 3,4,7T57 in the standard representation: together with the initial framework (top) and alone (bottom).

(ii) **Cluster representation.**

In the cluster representation the asymmetric (3,4)-coordinated Zn_2 dimer is considered a single 4-coordinated node, while the ligand can be mapped on two 3-coordinated nodes (fig. S34). The underlying net is then 3,3,4-coordinated **zyl** (fig. S35), which is a **lon-**derived net. The **zyl** is a uniform net with a point symbol of $(8^3)_2(8^6)$.

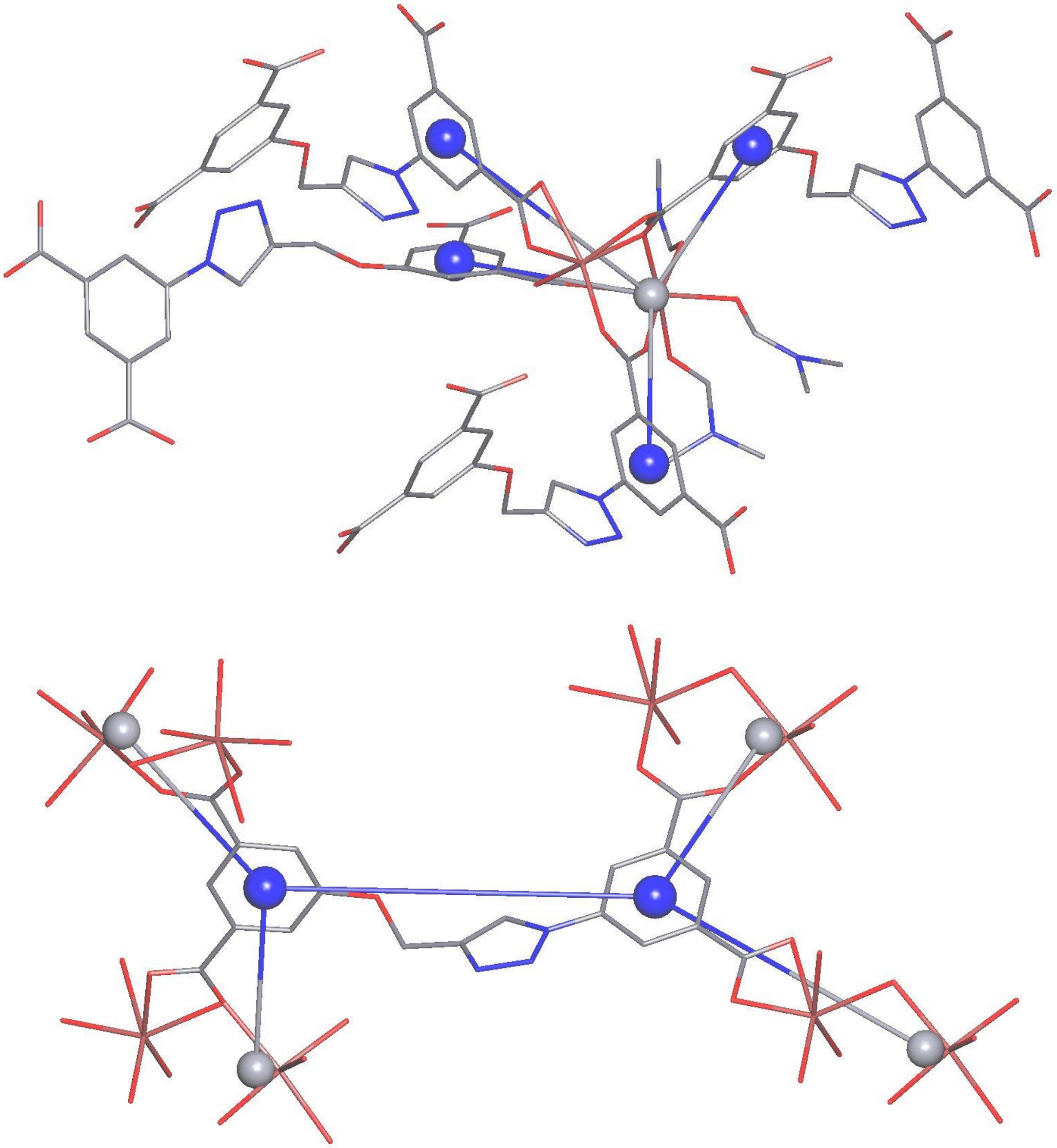

Figure S34. 4-coordinated dimer nodes (big grey balls) and the ligand as two 3-coordinated nodes (blue balls) in the cluster representation.

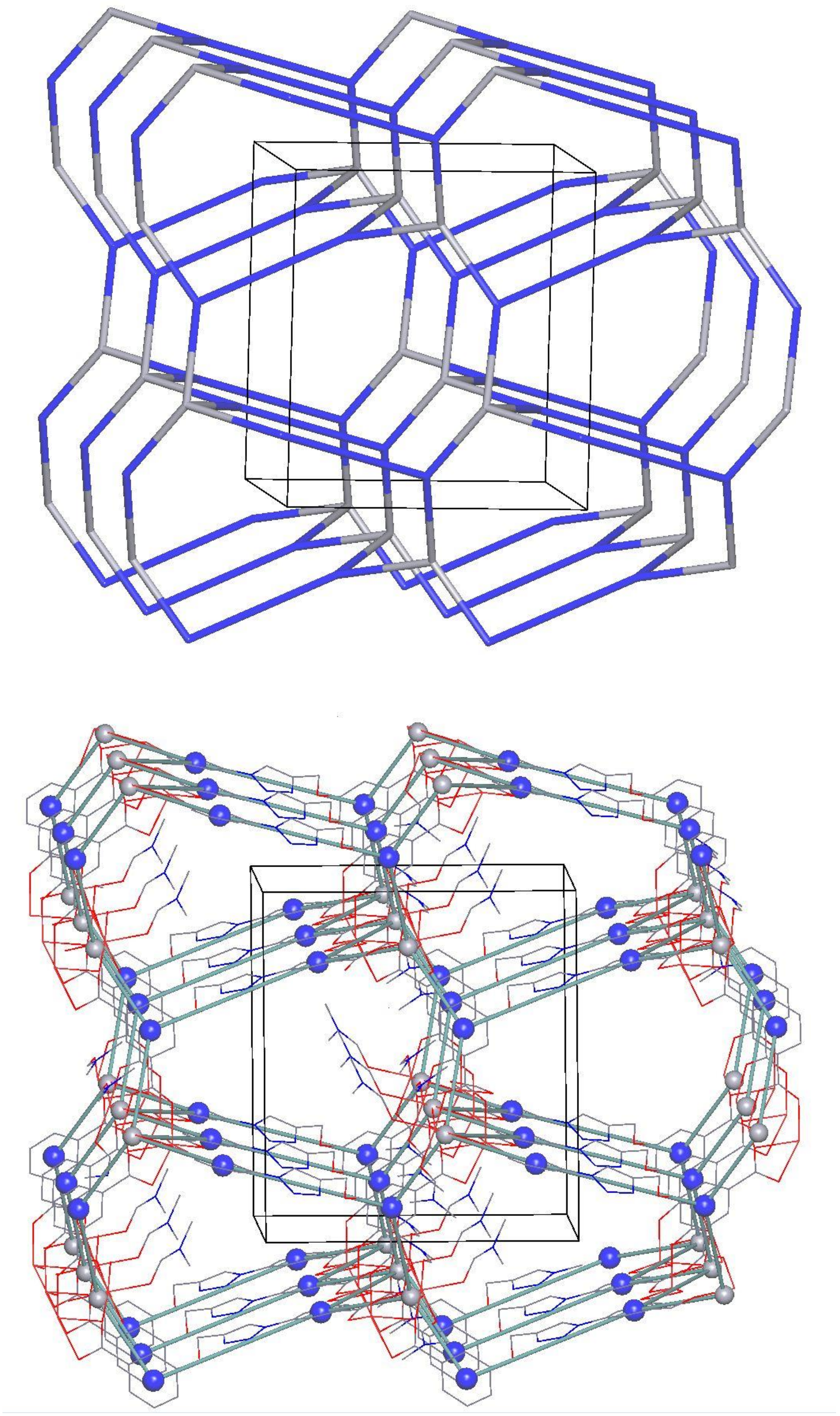

Figure S35. The underlying net in the cluster representation with the zyl topology, a lon-derived uniform net with Point Symbol $(8^3)_2(8^6)$ (top) and the net merged with the corresponding framework (bottom).

The asymmetric unit contains one L ligand (disordered over two positions) and two crystallographically independent Zn(II) ions with three coordinated DMF molecules. In the crystal

structure, typical Zn_2 asymmetric dimeric clusters are formed between two neighboring Zn(II) ions, which are bridged by two bidentately μ_2-η^1:η^1 bridging and one μ_2-η^2:η^1 chelating bridging carboxylate groups from three different L ligands, with a Zn-Zn distance of 3.4316(9) Å. The octahedral coordination of the Zn_2 cluster ions is completed by three terminal DMF molecules and one chelating carboxylate group from another symmetry equivalent L ligand, for the first and second Zn(II) ion, respectively. The framework can be described by single 4-coordinated nodes, corresponding to the asymmetric (3,4)-coordinated Zn_2 dimers, and the ligand, which can be mapped onto two 3-coordinated nodes. The underlying net shows then a 3,3,4-coordinated uniform **zyl** topology, which is a **lon**-derived net (Figures 36S and 37S).

The standard net topology is found 3,4,7- coordinated 3,4,7T57, with a point symbol of $(8^3)_2(8^6)$ for the net. The $Zn_2(CO_2)_4$ can be considered as SBU, while second SBU's are formed by the L^{4-} ligands. Here, the carboxylate groups of the L^{4-} ligands are more torsioned with respect to the phenyl rings (dihedral angles in the range of 0.4° to 15.7°), and, more strikingly, the ligand phenyl rings are highly torsioned (dihedral angle of 67.2°) with respect to each other. This arrangement features 2D grid-like solvent channels, built up by irregular solvent channels along the [100] direction (Figure 37S), with one of the coordinated DMF molecules pointing into the channels (~5.5 x 9.0 Å) and smaller elliptical cylindrical channels (~4.0 x 5.0 Å) along the [010] direction (taking into account the van der Waals radii) (Figure 37S). These solvent channels are further shown along the [001] direction in Figure 9 along different axis. The total potential solvent accessible void volume was calculated to be 966.5 $Å^3$ (39.4% of the unit cell volume) using PLATON.[4]

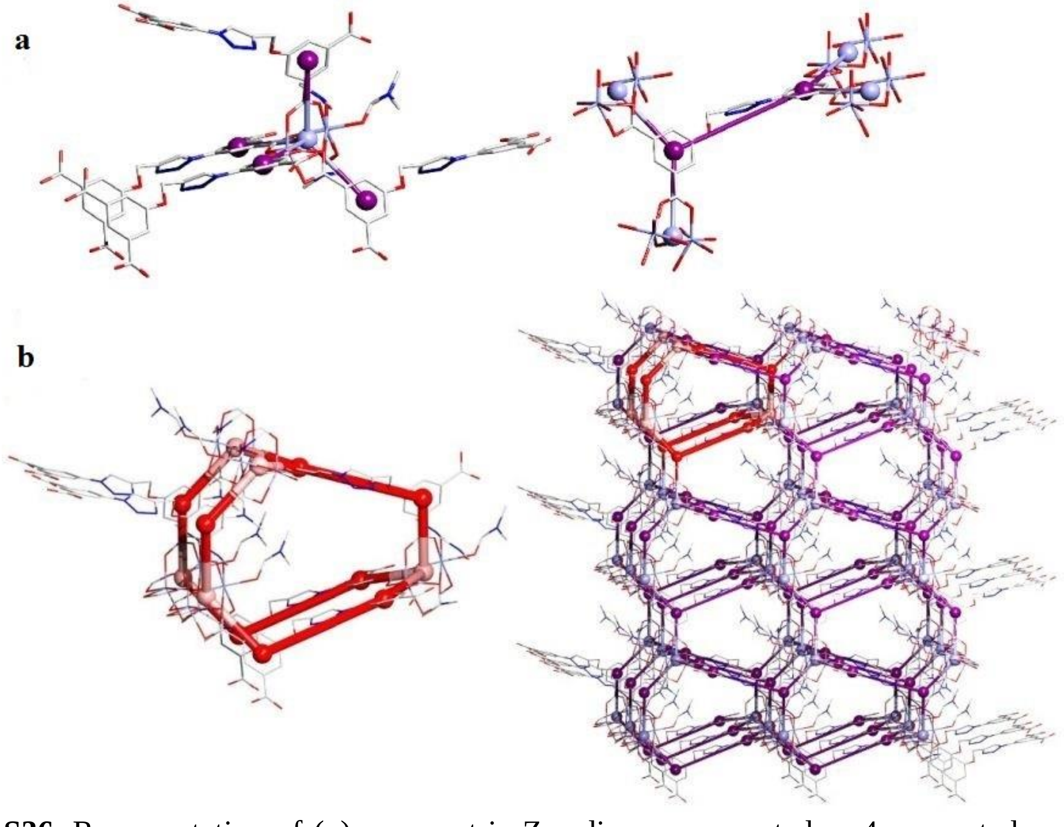

Figure S36. Representation of (**a**) asymmetric Zn_2 dimer represented as 4-connected node like tetrahedral $Zn_2(CO_2)_4$ SBU's (left) and rectangular L^{4-}- SBU's ligand as two 3-connected nodes;

(**b**) The underlying net in the cluster representation with the **zyl** topology, a **lon**-derived uniform net (left) and the net merged with the corresponding framework (right)."

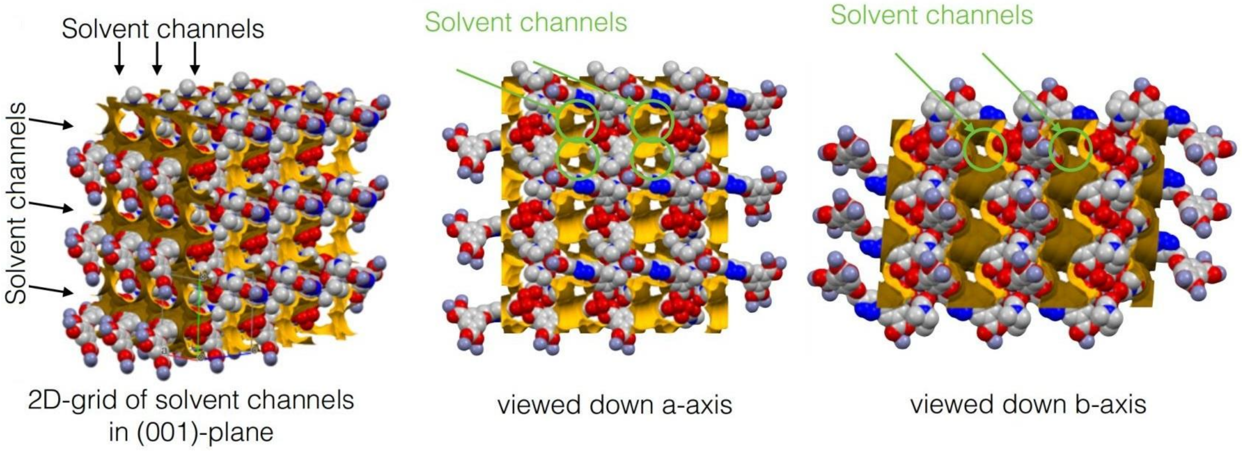

Figure S37. Packing in the structure of Zn-MOF, in ball-and-stick; packing diagrams showing smaller elliptical cylindrical channels along the [001] direction, respectively. The framework atoms are depicted in space-filling (VDW radii) representation. The DMF molecules are depicted in ball-and-stick representation. Hydrogen atoms are omitted for clarity.

9. FTIR and TGA of MOFs

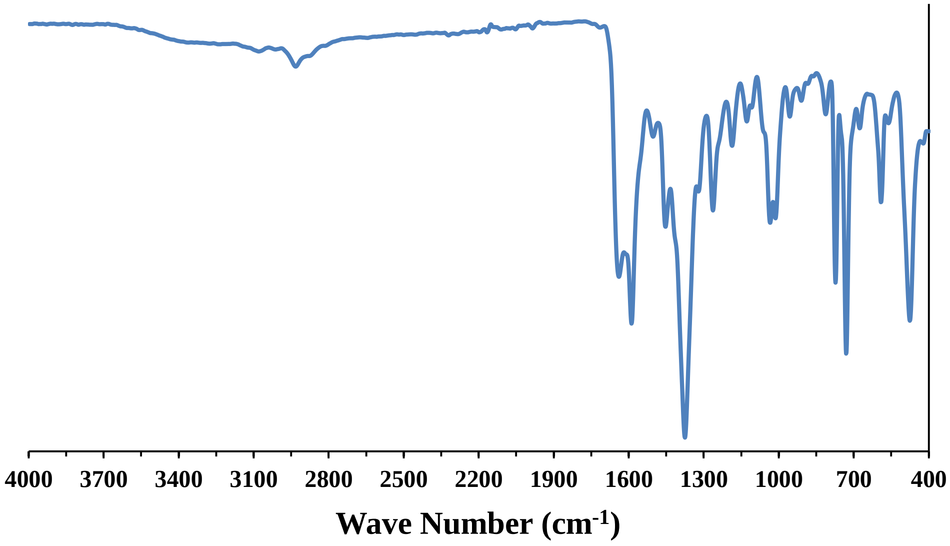

Figure S38. FTIR spectrum of the as-synthesized Cu-MOF.

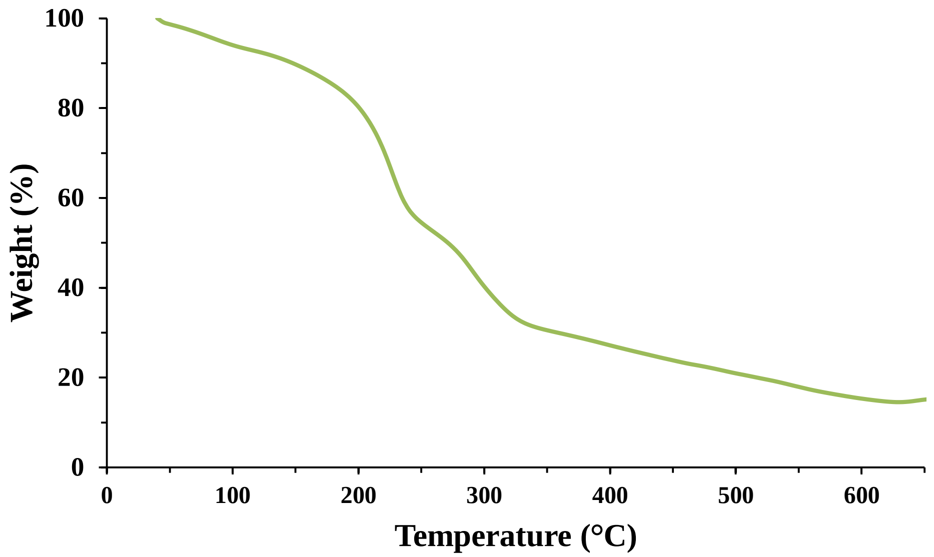

Figure S39. TG for the as-synthesized Cu-MOF.

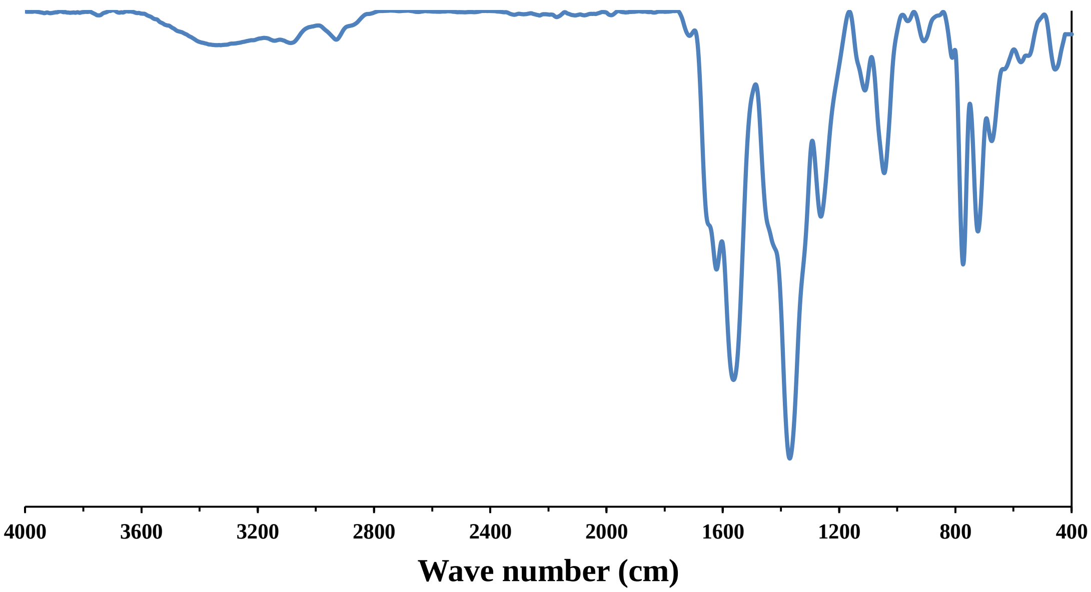

Figure S40. FTIR spectrum of the as-synthesized Zn-MOF.

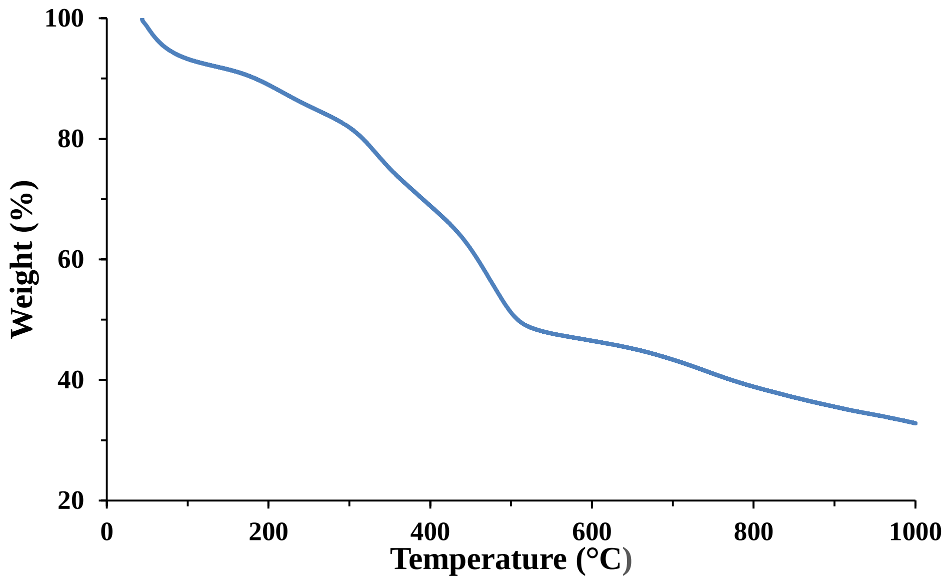

Figure S41. TG for the as-synthesized Zn-MOF.

10. Stability of the materials in buffer solution

The stability of the both types of materials *i.e.*, MOP polymeric network and MOF in buffer solution was checked. The materials were soaked in phosphate buffer solution (PBS) of pH 7.4 for one day followed by filtration, washing, and drying in vacuum oven at 80 °C overnight. UV/Vis spectra of the PBS filtrates collected after soaking Cu-MOF and CuMOPN1-(Cu) in PBS in comparison to blank PBS proved that in none of the tested materials Cu-Cu paddle-wheels or free copper were detected. Furthermore, FTIR spectra of the materials before and after treatment with buffer solution are identical which confirms the stability of these materials in PBS.

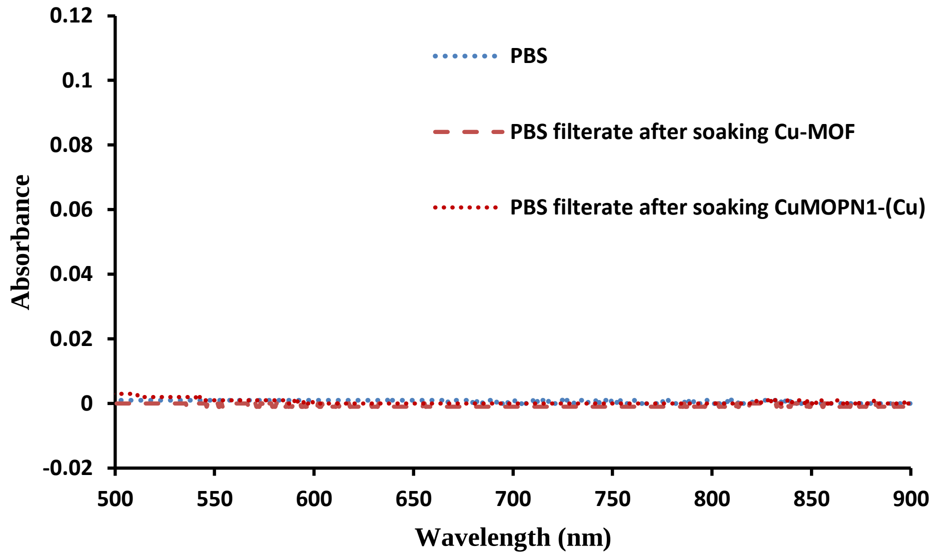

Figure S42. UV/Vis. spectra of the PBS filtrates collected after soaking Cu-MOF and CuMOPN1-(Cu) in PBS of pH 7.4 in comparison to blank PBS.

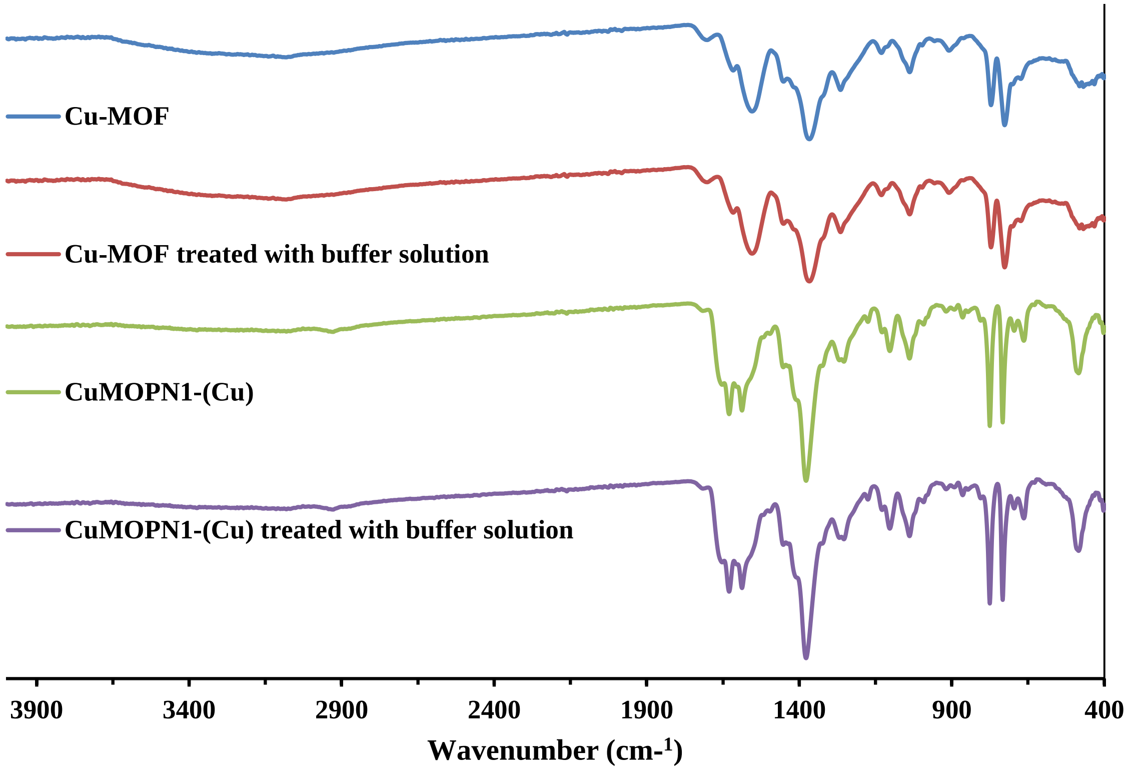

Figure S43. FTIR of the Cu-MOF and CuMOPN1-(Cu) before and after treatment with PBS of pH 7.4 (the materials were soaked for one day in PBS followed by filtration, washing and vacuum drying at 80 °C overnight).

References

1 Mallick, A., Garai, B., Díaz, D. D. & Banerjee, R. Hydrolytic conversion of a metal–organic polyhedron into a metal–organic framework. *Angew. Chem. Int. Ed.* **51**, 13755-13759 (2013).

2 Pierrat, P., Vanderheiden, S., Muller, T. & Brase, S. Functionalization of hexakis methanofullerene malonate crown-ethers: promising octahedral building blocks for molecular networks. *Chem. Commun.*, 1748-1750 (2009).

3 Dolomanov, O. V., Bourhis, L. J., Gildea, R. J., Howard, J. A. & Puschmann, H. OLEX2: a complete structure solution, refinement and analysis program. *J. Appl. Crystallogr.* **42**, 339-341 (2009).

4 Sheldrick, G. A short history of SHELX. *Acta Crystallogr. A* **64**, 112-122 (2008).

5 Spek, A. Structure validation in chemical crystallography. *Acta Crystallogr. D* **65**, 148-155 (2009).

6 Blatov, V. A., Shevchenko, A. P. & Proserpio, D. M. Applied topological analysis of crystal structures with the Program Package ToposPro. *Cryst. Growth Des.* **14**, 3576-3586 (2014).

7 O'Keeffe, M., Peskov, M. A., Ramsden, S. J. & Yaghi, O. M. The reticular chemistry structure resource (RCSR) database of, and symbols for, crystal nets. *Acc. Chem. Res.* **41**, 1782-1789 (2008).

8 Alexandrov, E., Blatov, V., Kochetkov, A. & Proserpio, D. Underlying nets in three-periodic coordination polymers: topology, taxonomy and prediction from a computer-aided analysis of the Cambridge Structural Database. *CrystEngComm* **13**, 3947-3958 (2011).
